# Supplementary material for: Membrane-Wrapping Contributions to Malaria Parasite Invasion of the Human Erythrocyte
Source: Biophys J. 2014 Jul 1;107(1):43–54. doi: 10.1016/j.bpj.2014.05.024 (PMC4184798; doi:10.1016/j.bpj.2014.05.024)
Supplement: Document S1. Four figures, supporting data, some details of the theoretical analysis, a discussion of discontinuous transitions in the phase diagram, and reference 79 [file mmc1.pdf]

Supporting Material

Membrane-wrapping contributions to malaria parasite invasion of the human erythrocyte

Sabyasachi Dasgupta [1], Thorsten Auth [1], Nir S. Gov [2,3], Timothy J. Satchwell [4], Eric Hanssen [5], Elizabeth S. Zuccala [6,7], David T. Riglar [6,7], Ashley M. Toye [4,8], Timo Betz [3], Jake Baum [6,7,9]* and Gerhard Gompper [1]*

DESCRIPTION OF ERYTHROCYTE-MEMBRANE WRAPPING

Towards calculating membrane-wrapping contributions to invasion we sought to identify core parameters associated with parasite entry. Four key parameters were selected, adhesive forces, bending energy, target membrane tension and a line tension (Fig. S1).

When the host membrane (Fig. S1*A*, orange) comes close to the parasite membrane (Fig. S1*A*, blue), adhesion molecules form an attractive interaction between the two membranes (illustrated with red springs). The adhesion strength, wH_0, which quantifies the adhesion energy, depends on the local concentration of the adhesion molecules. In experiments, close contact between the merozoite and the erythrocyte membrane is observed [1] that supports a close adhesive interaction between the two cells (Fig. S2*A*).

Bending a membrane also requires energy that is determined by two contributions; the bending modulus κ, which reflects the rigidity of the membrane against a forced bending, and the actual degree of bending which is quantified by the curvature C (Fig. S1*B*). The curvature C can be simply visualized by fitting a circle along the curve and then taking the inverse radius of this circle: $C = 1/R$. Hence, high curvature corresponds to a small circle radius, while a flat membrane has no curvature, which corresponds to an infinite radius. Since the membrane is a two-dimensional $(2D)$ surface, curvature can exist at any point in the two directions. The relevant curvature is the mean curvature, $H = (1/R_x + 1/R_y)/2$.

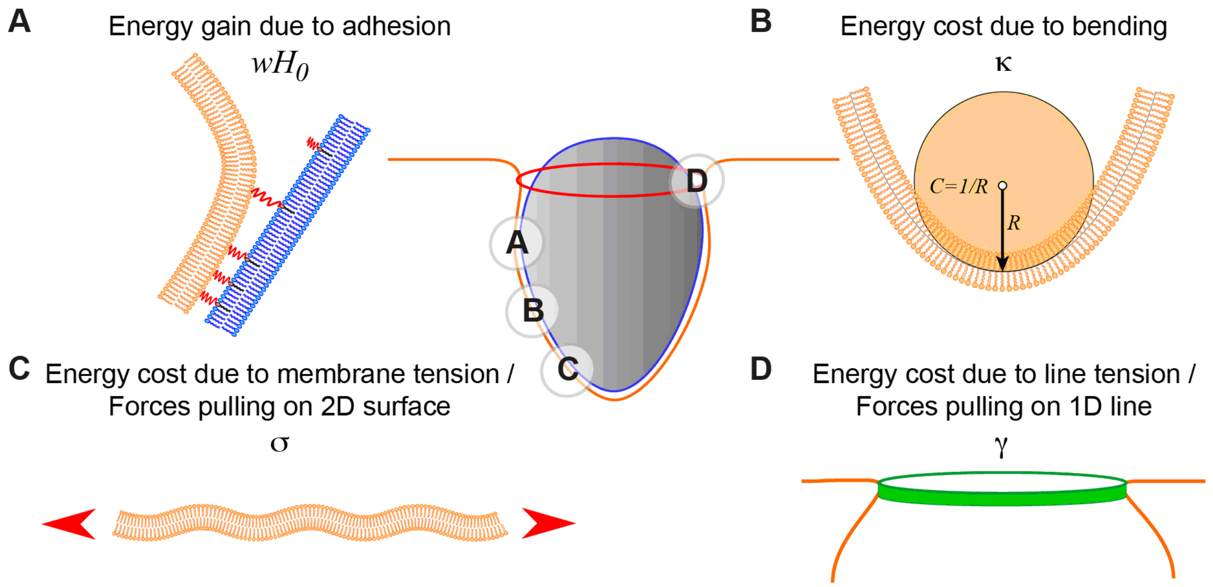

Figure S1. Schematic explanation of the energetic contributions used to model the role of the host membrane in merozoite invasion
The energetic contributions to membrane wrapping in our model are characterized by **A.** adhesion strength, **B.** bending rigidity, **C.** membrane tension, and **D.** line tension (see main text for details).

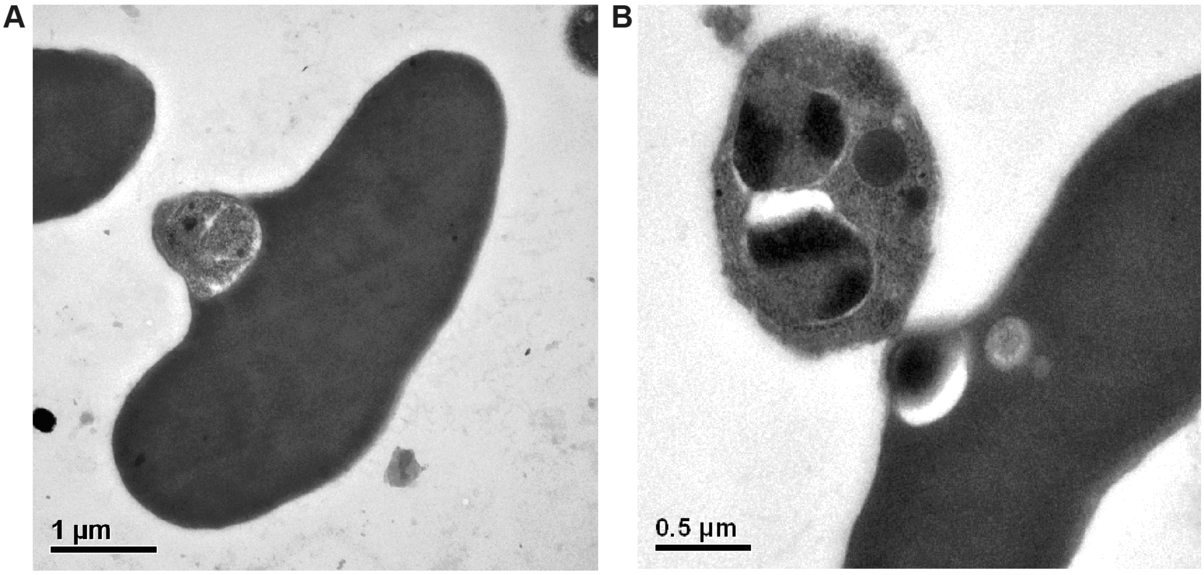

Figure S2. Electron micrographs of merozoites from *Plasmodium falciparum* in the process of invading an erythrocyte.
A. Demonstration of the close proximity between plasma membrane of the merozoite and the plasma membrane of the erythrocyte during invasion as revealed by high pressure freezing and freeze substitution (as compared with other approaches which can give rise to a substantial slack space between parasite and host cell in nascent parasitophorous vacuole). Imaging conditions as described in Ref. [1]. **B.** Release of membrane from apex of parasite during the invasion process. Here merozoite invasion is arrested with cytochalasin D (an inhibitor of invasion), which prevents completion of invasion but does not prevent release of membrane whorls from the rhoptry bulb. Imaging conditions as described in Ref. [1].

Two tension forces play key roles in the invasion process. Host membrane tension relates to an energetic cost that is related to a lateral pulling on the membrane (Fig. S1*C*). Typically, biological membranes are soft enough to fluctuate because of thermal movement, which is analogous to the Brownian diffusion of a small particle. This movement means that the apparent membrane area measured when looking on the membrane is smaller than the real area that includes the area "hidden" in the fluctuations. Physically, it means that when a pulling force is applied (red arrows), the apparent membrane area increases. The membrane tension can also be reduced by release of membrane from the apex of the parasite (Fig. S2*B*). The membrane tension is then defined by the force required to increase the membrane area. Hence high tension (represented by the term σ) means that high forces are required to increase the apparent area.

A final energetic contribution comes from the spring-like behaviour of the cytoskeleton along the furrow at the membrane inclusion, referred to here as a line tension (Fig. S1*D*). This spring-like behaviour can be modelled by an elastic rubber band that lies around the furrow, and hence wants to constrict the furrow. The spring stiffness of this rubber band is modelled by the line tension (represented by the term γ). Physically, in the first phase on invasion (PW I) the merozoite has to stretch this rubber band, which costs energy. However, once over the point of maximal diameter (PW II), the line tension actually helps pushing the merozoite inside the host cell.

CALCULATION OF THE MEROZOITE WRAPPING ENERGY

The key ingredients for calculating the energy for wrapping a merozoite are the shape of our archetypal merozoite and our model for the deformation energy defined by Eq. 2 of the main text. The egg shape of the archetypal merozoite is $(x^2 + y^2 + z^2)^2 = R_a x^3 + (R_a - R_b)x(y^2 + z^2)$ with $R_a = 1\,\mu m$ and $R_b = 0.7\,\mu m$. For the calculation of the deformation energy, the shape is parametrized in spherical coordinates using the polar angle u and the azimuthal angle v,

$$x(u,v) = \left[\frac{(2R_a - R_b)\,sin\,(v)}{4} + \frac{R_b\,sin\,(2v)}{8}\right]\cos\,(u)\,, \tag{1}$$

$$y(u,v) = \left[\frac{(2R_a - R_b)\,sin\,(v)}{4} + \frac{R_b\,sin\,(2v)}{8}\right]\sin\,(u)\,,$$

$$z(u,v) = \left[\frac{2R_a + R_b\,cos\,(v)}{4}\right]\cos\,(v)\,,$$

where $0 \le u < 2\pi$ and $0 \le v < \pi$. The distance from the symmetry axis as function of the azimuthal angle is

$$\rho(v) = \sqrt{x^2 + y^2} = \left[\frac{(2R_a - R_b)\,sin\,(v)}{4} + \frac{R_b\,sin\,(2v)}{8}\right] \tag{2}$$

and the radial coordinate of the merozoite surface,

$$r(v) = \sqrt{x^2 + y^2 + z^2}$$

$$= \sqrt{\left[\frac{(2\,R_a - R_b)\,sin\,(v)}{4} + \frac{R_b\,sin\,(2v)}{8}\right]^2 + \left[\frac{2R_a + R_b\,cos\,(v)}{4}\right]^2} \tag{3}$$

In spherical coordinates, Eq. 2 in the main text becomes

$$\mathcal{E} = 2\pi \int_0^{v_{\mathrm{wrap}}} d\,v\,g_{\rho v}\,\rho(v)\,[2\kappa(H(v))^2 + \sigma - wH(v)] + 2\pi\rho(v)\gamma\,, \tag{4}$$

where $g_{\rho v} = \sqrt{(d\rho^2 + dv^2)} = (2R_a - R_b)\sin\,(v)/4 + R_b \sin\,(2v)/8$, and v_{wrap} determines the degree of wrapping. Note that in our model the membrane deformation energy is only calculated for the membrane that is attached to the merozoite. The integrals are evaluated numerically using the computer algebra program *Maple*.

Both the bending energy and the adhesion energy contributions in our model require the calculation of the mean curvature. The mean curvature can be expressed by the fundamental forms of the merozoite surface [2],

$$H = \frac{eG - 2fF - gE}{EG - F^2}\,, \tag{5}$$

where E, F, and G are the first fundamental forms and e, f, and g are the second fundamental forms.

DISCONTINUOUS PHASE TRANSITIONS WITH ENERGY BARRIERS

The phase boundary W_0 between the NW and the PW states in Fig. S3 and Fig. 4 in main text is the binding transition that we use to discuss reorientation based on the local adhesion strength and bending energy. With a small membrane and line tension, PW and even CW regions may extend to smaller adhesion strengths than those associated with the threshold adhesion strength for W_0. This might at first appear to be surprising, since for very shallow wrapping the value of the membrane tension would not be expected to alter the transition [3,4]. However, the nature of the W_0 transition and the W_1, W_2, and E transitions in the phase diagram are different: while the W_0 transition is a continuous transition, the transitions W_1, W_2 and E are associated with an energy barrier (E also indicates when the unwrapped and the completely-wrapped state have equal energy). These latter transitions correspond to a jump in the fraction of the merozoite surface that is in contact with the enveloping membrane as the transition is passed (Fig. 4). For adhesion strengths very close to those phase boundaries, transitions cannot occur spontaneously.

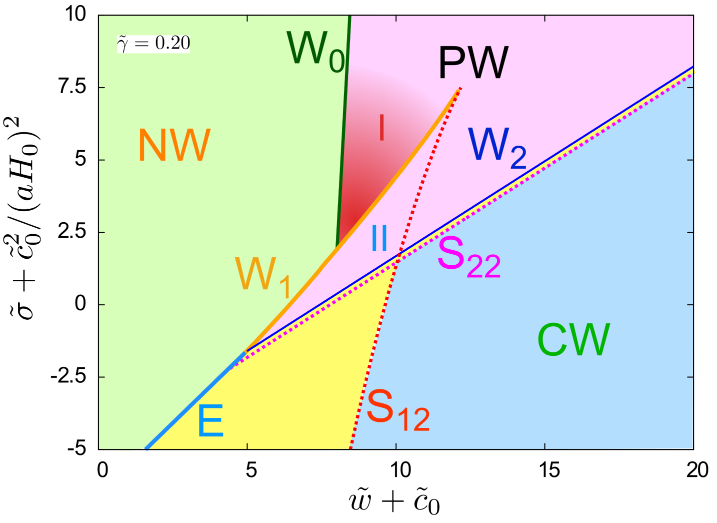

A.

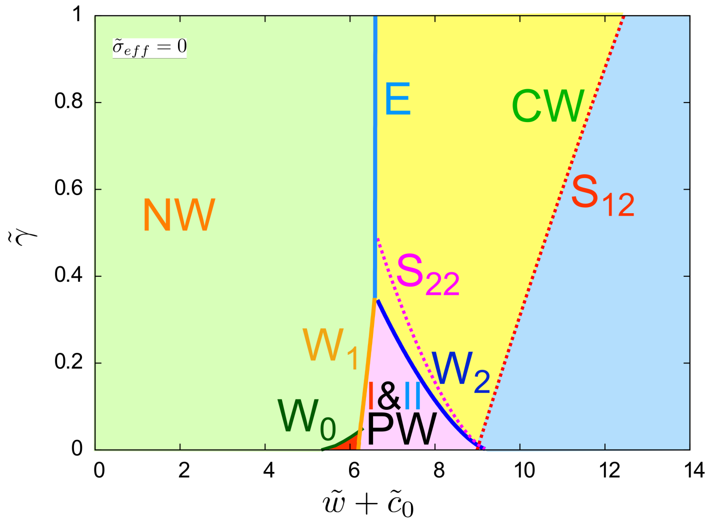

B.

Figure S3. Phase diagrams for a tip-first oriented merozoite.
Phase diagrams Fig. 4 in the main text including the upper spinodals. S_{12} indicates the adhesion strengths at which the transition associated with the phase boundary W_1/E occurs spontaneously, while the spinodal S_{22} belongs to the phase boundary W_2. Spontaneous wrapping therefore occurs in the blue part of the CW region, while in the yellow part an energy barrier has to be overcome. **A.** Wrapping states for fixed reduced line tension $\tilde{\gamma} = 0.20$ and several values of adhesion strength and membrane tension. **B.** Wrapping states of the system of a tip-first oriented merozoite for vanishing effective membrane tension and several values of adhesion strength and line tension.

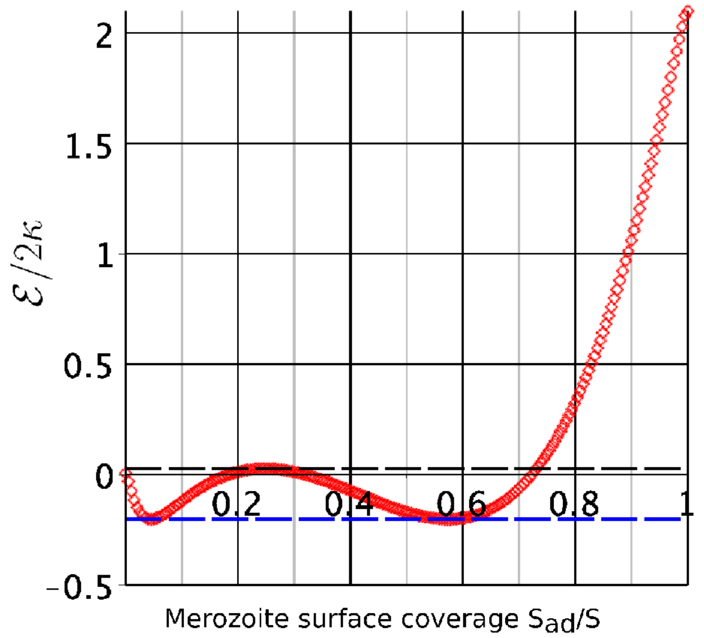

Figure S4. Energy profile for a state on the W1 phase boundary.
Energy profile at reduced effective adhesion strength $\tilde{\boldsymbol{w}}_{\boldsymbol{eff}} = \mathbf{10.066}$ and reduced membrane tension $\tilde{\boldsymbol{\sigma}} = \mathbf{4.5}$, see Figs. 4 and 5 in main text. The transition between the PW I state for $\boldsymbol{S_{ad}/S} = \mathbf{0.045}$ and the PW II state for $\boldsymbol{S_{ad}/S} = \mathbf{0.575}$ is associated with an energy barrier $\boldsymbol{\Delta\mathcal{E}/2\kappa} = \mathbf{0.228}$ whose maximum is found at $\boldsymbol{S_{ad}/S} \approx \mathbf{0.25}$.

For the phase boundaries W_1 and W_2, the spinodals S_{12} and S_{22} indicate the parameter regime where the transition occurs spontaneously and beyond that the energy barrier vanishes with increasing $\tilde{w} + \tilde{c}_0$. Only for values of adhesion strength that are larger than those of the spinodals does the energy barrier vanish and further wrapping occur spontaneously. In Fig. 5 in main text, we indicate the wrapping fractions of the stable states between which the discontinuous transitions occur; the tie lines mark the parameter region that corresponds to states on the energy barrier. In Fig. S4 we show the energy profile for a state on the discontinuous transition W1 when the system traverses from a low wrapping fraction (PW I) to a higher wrapping fraction (PW II). The profile shows explicitly the energy barrier that needs to be overcome for further wrapping at the adhesion strength $\tilde{w} = 10.066$ for reduced membrane tension $\tilde{\sigma} = 4.5$. The low wrapping fraction state is found at $S_{ad}/S = 0.045$ and the high wrapping fraction state at $S_{ad}/S = 0.575$. The maximum of the energy barrier with height $\Delta\mathcal{E}/2\kappa = 0.228$ is located at $S_{ad}/S \approx 0.25$. In order to jump from the PW I state to a PW II state, there is thus a need for an “activation energy” that facilitates invasion. The energy barrier for the W_2 transition that separates the partially-wrapped state with high wrapping fraction from the completely-wrapped state is typically much smaller than the energy barrier for the W_1 transition. Hence, the spinodal is found for adhesion strengths close to values where the W_2 transition occurs. Actomyosin motor activity may be a key determinant to overcome wrapping energy barriers and the barriers can be used to estimate the required motor activity. However, activity alone is not sufficient to study invasion. When the motor stops to act (e.g. once invasion is almost complete) the merozoite will only remain wrapped in cases where its final state lies within the CW region of the phase diagram (Fig. S3 and Fig. 4 in main text).

Supporting References

1. Hanssen E., Dekiwadia C., Riglar D. T., Rug M., Lemgruber L., Cowman A. F., Cyrklaff M., Kudryashev M., Frischknecht F., Baum J. and Ralph S. A. 2013. Electron tomography of Plasmodium falciparum merozoites reveals core cellular events that underpin erythrocyte invasion. *Cell Microbiol*. **15**: 1457-1472.
2. Elsa A., Salamon S. and Gray A. 2006. *Modern differential geometry of curves and surfaces with Mathematica*. CRC Press.
3. Lipowsky R. and Döbereiner H. G. 1998. Vesicles in contact with nanoparticles and colloids. *Europhysics Letters*. **43**: 219.
4. Farago O. and Pincus P. 2004. Statistical mechanics of bilayer membrane with a fixed projected area. *Journal of Chemical Physics*. **120**: 2934.
