# Supplementary material for: Membrane-Wrapping Contributions to Malaria Parasite Invasion of the Human Erythrocyte
Source: Biophys J. 2014 Jul 1;107(1):43–54. doi: 10.1016/j.bpj.2014.05.024 (PMC4184798; doi:10.1016/j.bpj.2014.05.024)
Supplement: Document S2. Article plus Supporting Material [file mmc2.pdf]

Article

Membrane-Wrapping Contributions to Malaria Parasite Invasion of the Human Erythrocyte

Sabyasachi Dasgupta,[1] Thorsten Auth,[1] Nir S. Gov,[2,3] Timothy J. Satchwell,[4] Eric Hanssen,[5] Elizabeth S. Zuccala,[6,7] David T. Riglar,[6,7] Ashley M. Toye,[4,8] Timo Betz,[3] Jake Baum,[6,7,9,*] and Gerhard Gompper[1,*]

[1]Institute of Complex Systems and Institute for Advanced Simulation, Forschungszentrum Jülich, Jülich, Germany; [2]Department of Chemical Physics, Weizmann Institute of Science, Rehovot, Israel; [3]Centre de Recherche, Institut Curie, Paris, France; [4]School of Biochemistry, University of Bristol, Bristol, United Kingdom; [5]Advanced Microscopy Facility, Bio21 Molecular Science and Biotechnology Institute, University of Melbourne, Parkville, Victoria, Australia; [6]Division of Infection and Immunity, Walter and Eliza Hall Institute of Medical Research, Parkville, Victoria, Australia; [7]Department of Medical Biology, University of Melbourne, Parkville, Victoria, Australia; [8]Bristol Institute for Transfusion Sciences, NHS Blood and Transplant, Bristol, United Kingdom; and [9]Department of Life Sciences, Imperial College London, South Kensington, London, United Kingdom

ABSTRACT The blood stage malaria parasite, the merozoite, has a small window of opportunity during which it must successfully target and invade a human erythrocyte. The process of invasion is nonetheless remarkably rapid. To date, mechanistic models of invasion have focused predominantly on the parasite actomyosin motor contribution to the energetics of entry. Here, we have conducted a numerical analysis using dimensions for an archetypal merozoite to predict the respective contributions of the host-parasite interactions to invasion, in particular the role of membrane wrapping. Our theoretical modeling demonstrates that erythrocyte membrane wrapping alone, as a function of merozoite adhesive and shape properties, is sufficient to entirely account for the first key step of the invasion process, that of merozoite reorientation to its apex and tight adhesive linkage between the two cells. Next, parasite-induced reorganization of the erythrocyte cytoskeleton and release of parasite-derived membrane can also account for a considerable energetic portion of actual invasion itself, through membrane wrapping. Thus, contrary to the prevailing dogma, wrapping by the erythrocyte combined with parasite-derived membrane release can markedly reduce the expected contributions of the merozoite actomyosin motor to invasion. We therefore propose that invasion is a balance between parasite and host cell contributions, evolved toward maximal efficient use of biophysical forces between the two cells.

INTRODUCTION

The asexual cycles of infection, through replication, rupture, and reinfection of human erythrocytes by *Plasmodium* parasites are responsible for all malaria disease pathology. Extensive effort has focused on understanding the cellular and molecular basis for each stage of the process, invasion in particular, with a view to designing novel chemotherapeutics or vaccines to prevent or treat the disease (1). Invasion of the erythrocyte itself is mediated by the blood stage parasite, called the merozoite, which is thought to use an internal molecular motor based on actin and myosin to drive itself into the red cell (2). Conceptually, the process can be divided into discrete steps, defined by a range of imaging studies using electron (3–5), tomographic (6), fluorescence (7–10), and video microscopy (11,12) as shown schematically in Fig. 1. Invasion commences with low affinity, long-range (12 to 40 nm), and nondirectional binding of the erythrocyte by the merozoite, which then reorients such that the merozoite apex directly contacts the target cell.

Formation of a close-range interaction follows (4 nm or less), leading to the establishment of an erythrocyte-merozoite tight junction (3,4). This critical structure, seen as an electron dense zone between erythrocyte and merozoite by electron microscopy, is the organizing nexus around which invasion events appear to be orchestrated. It acts as the aperture through which the merozoite passes during invasion and segregates erythrocyte membrane from an emerging vacuolar membrane (likely parasite membrane-derived in part), which fuses to form the parasitophorous vacuole into which the parasite moves and develops postinvasion (see (13,14), and references therein). Each of these steps is facilitated by an array of merozoite surface proteins (MSPs), which permanently pattern the parasite surface, and apically secreted parasite antigens, released at egress from the infected cell before reentry. Many of the latter group are lead candidates for inclusion in developmental blood stage vaccines, including the apical membrane antigen (AMA)-1, erythrocyte binding antigens, and reticulocyte binding antigen homolog (Rh) proteins, which interact to varying degrees, and at varying distances, with erythrocyte membrane components (1).

Several studies have attempted to map out a broad model of invasion, incorporating the breadth of molecular and

Submitted December 16, 2013, and accepted for publication May 19, 2014.

*Correspondence: g.gompper@fz-juelich.de or jake.baum@imperial.ac.uk

This is an open access article under the CC BY license (http://creativecommons.org/licenses/by/3.0/).

Editor: Tobias Baumgart.

© 2014 The Authors

0006-3495/14/07/0043/12 $2.00

http://dx.doi.org/10.1016/j.bpj.2014.05.024

CrossMark

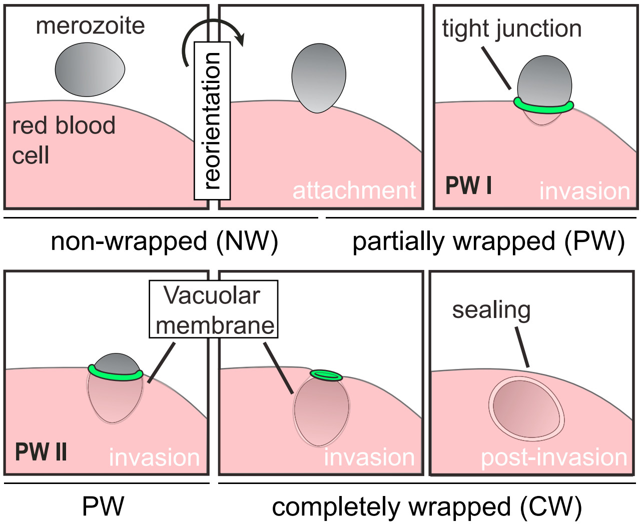

FIGURE 1 The stages of merozoite invasion. Schematic representation depicting different wrapping phases of the merozoite from reorientation through to invasion and postinvasion (see below for definitions of wrapped states). To see this figure in color, go online.

cellular events (e.g., (7,9,10)). However, few studies have taken into consideration the biophysical interactions between host and parasite cells, in particular the contribution that the erythrocyte membrane and underlying cytoskeleton might play (15). This has largely been influenced by long-standing evidence that activity of the parasite actomyosin motor alone defines successful host-cell entry (16,17). Surprisingly, such a parasite-centric model is quite unique among human intracellular pathogens, which almost universally employ a degree of host involvement in invasion (18). Instead, its broad acceptance relies heavily on the general perceived inactivity of the mature erythrocyte (19) and studies using the related apicomplexan parasite, *Toxoplasma gondii*, which, until recently, was believed to invade independently of host-cell remodeling processes (20). Recently, this view has started to be challenged by studies showing that host cell cytoskeletal rearrangements do occur during *Toxoplasma* and nonerythroid *Plasmodium* invasion (21) and the recent demonstration of a residual level of invasion in the absence of myosin and actin in *Toxoplasma* (22). These studies clearly suggest that a role of processes other than motor-driven force production in facilitating apicomplexan invasion deserves focused attention.

Certainly, there is a body of evidence that the erythrocyte responds, at least minimally, to invasion both physically, with the membrane oscillating or flexing (11–13), and biochemically (reviewed in (19)). The physical response, with visible folds on parasite binding, is expected for a membrane that has a shear modulus (23,24), which is caused by the regular spectrin network that supports the erythrocyte membrane (25,26). Biochemical contributions have also been widely studied, and hint at a potential membrane contribution to the parasitophorous vacuole (14). To date, however, no strong evidence exists to suggest an energetic contribution to invasion from the erythrocyte.

Here, we have sought to assess the energetic contributions of the *Plasmodium* parasite and wrapping by the erythrocyte and parasite-derived membranes to invasion. In contrast to previous membrane wrapping calculations that have explored models involving spherical, ellipsoidal, or rod-like particles (27–34), we have uniquely incorporated the asymmetrical egg-like shape of the merozoite, which influences differentially wrapped states. Using numerical calculations of membrane interactions and membrane wrapping processes between an experimentally determined archetypal egg-shaped merozoite and the erythrocyte, we present evidence that membrane wrapping of the erythrocyte can account for merozoite reorientation to its apex in an entirely parasite-energy independent manner. Furthermore, a considerable portion of the energy requirements for subsequent stages of full parasite invasion (i.e., for complete membrane wrapping) can also be achieved through parasite-induced modifications to the erythrocyte membrane and by parasite-injected membrane material that may alter surface tension and spontaneous curvature of the wrapping membrane. Wrapping under these conditions requires only a small energetic input from the parasite actomyosin motor for entry, i.e., overcoming energy barriers between stable membrane-wrapped states. Seen in this light, we propose that erythrocyte invasion should be considered as evolved toward a state of maximal energetic efficiency, exploiting both innate host-cell properties and parasite motor force to facilitate complete entry.

Our article is organized as follows. We first use cryo-x-ray tomography to construct an idealized merozoite that approximates the experimentally observed merozoite shape. We then calculate the energetic contributions of membrane wrapping to merozoite reorientation and subsequent invasion into the erythrocyte, using a model with bending-rigidity and membrane-tension contributions for the curvature elastic energy of the erythrocyte membrane, an adhesive interaction between the merozoite and the erythrocyte, and a line tension for the tight junction. For several parameter values in our theoretical model, we quantify the required motor force for invasion. Finally, we summarize our complete biophysical model for merozoite invasion assessing likely contributions of the actomyosin motor of the merozoite and wrapping-energy contributions of the erythrocyte membrane.

MATERIALS AND METHODS

Experimental derivation of merozoite dimensions

The culture of *Plasmodium falciparum* parasites using donated blood from the Australian Red Cross Society has been approved by The Walter and Eliza Hall Institute Human Ethics (HEC 86/17) Committee. *P. falciparum* parasites (from a D10 parental strain (35)) were maintained using standard culturing procedures in human O+ erythrocytes at 4% hematocrit with 0.5% wt/vol Albumax II (Life Technologies, Grand Island, NY). Cultures were maintained in synchrony using 5% Sorbitol treatment or via treatment

with 30 infectious units (~230 μg/mL) heparin (Pfizer, New York, NY) (35), and cultured through to schizogony for merozoite isolation. Free merozoites were filtered through a 1.2 μm, 32 mm syringe filter (Sartorius Stedim Biotech, Epsom, UK) as described (35), and then cryopreserved for x-ray analysis and imaged as detailed previously (6). Merozoite dimensions, volume, and surface area were calculated on rendered tomographic images following segmentation and alignment with IMOD with rendering via Blender (www.blender.org).

Deformation energy calculations and dimensionless parameters for erythrocyte-membrane wrapping

Toward calculating the energetic costs required to deform the erythrocyte membrane sufficiently to facilitate complete invasion, we decoupled the invasion process into two critical energetic steps (see Fig. 1): i), reorientation of the merozoite toward its apex (the site at which adhesive proteins are released and the required direction for successful invasion (11)); and ii), invasion itself (movement through the tight junction into the erythrocyte (3)). The physics of wrapping that characterizes the adhesion contribution to both reorientation and invasion is governed by bending energy and tension of the erythrocyte membrane, the contact energy between merozoite surface and erythrocyte membrane, and the line tension at the position of the tight junction where the merozoite squeezes through. Thus, the total energy required is

$$\mathcal{E} = \mathcal{E}_{bending} + \mathcal{E}_{membrane\ tension} + \mathcal{E}_{adhesion} + \mathcal{E}_{line\ tension}. \quad (1)$$

To calculate the total energy for the erythrocyte with the adhered merozoite, bending rigidity and membrane tension, adhesion strength, and line tension contributions are integrated over the entire membrane area, $S_{erythrocyte}$, the adhered membrane area, $S_{adhered}$, and the length of the contact line where the erythrocyte membrane detaches from the merozoite, respectively, so that

$$\mathcal{E} = \int_{S_{erythrocyte}} dS\, 2\kappa(H - c_0)^2 + \sigma \int_{S_{erythrocyte}} dS - w \int_{S_{adhered}} dS\, H + \gamma \int_{contact\ line} dl. \quad (2)$$

The various contributions to Eq. 2 are explained in more detail below. We calculate the energy on the parasite surface only assuming the outer membrane to be flat, i.e., we employ a cap-like model analogous to the model used in (36). We do not account for a direct contribution of the shear modulus of the red blood cell membrane, because we assume that successful invasion requires a destruction of the cytoskeleton on the membrane that wraps the merozoite. However, in our model the cytoskeleton around the merozoite remains intact and contributes to the line tension γ. Details of the numerical calculations are described in the Supporting Material.

Membrane wrapping of the merozoite can be understood as a competition between two energetic contributions: the elastic deformation energy of the membrane adhered to the merozoite and the specific contact interaction between merozoite and membrane. Note that the vacuolar membrane enveloping the merozoite after successful invasion is likely composed of both erythrocyte membrane and parasite-derived vacuolar membrane (added differentially during the stages of invasion stage). A key determinant for the membrane model is the curvature elasticity of the erythrocyte membrane, with bending rigidity κ, where the energy required for bending the membrane is determined by the squared mean curvature H^2 of the membrane at every point (37). The mean curvature is $H = (c_1 + c_2)/2$, with c_1 and c_2 being the principal curvatures corresponding to the maximum and minimum curvatures at each point of the membrane (38). The preferred average shape of the membrane is characterized by its spontaneous curvature c_0. A finite value for c_0 indicates that either the membrane or its surrounding is asymmetric and that consequently the preferred shape of the membrane is not flat, a feature clearly applicable to the erythrocyte (39,40). The bending energy is complemented by an energetic cost for the excess membrane area characterized by the membrane tension σ. This excess area can either be attributed to flattening out part of the intrinsic membrane fluctuations (41,42) or to other mechanisms. The tension term contains a contribution from the spectrin network that is adsorbed to the membrane (26,43).

For the merozoite to successfully enter the erythrocyte, the energy gain due to the contact of merozoite surface and the enveloping membrane must be sufficiently large, such that the completely wrapped state corresponds to the lowest energy. Furthermore, wrapping alone also requires a downhill pathway in the energy landscape, but—as discussed later—the actomyosin motors of the parasite may help to overcome energy barriers. A measure for both nonspecific adhesion and receptor binding is given by the adhesion strength w. Individual protein-mediated adhesion may couple to the membrane shape via membrane proteins that prefer curved regions (44–47), and we therefore assume in our model that the adhesion strength is dependent on the mean local membrane curvature H (if not stated otherwise). This also allows us to implement higher adhesion strengths at the tip-shaped apex of the merozoite, which accounts for the secretion of adhesion molecules from this region of the parasite (see description below). However, other distributions of receptors and thus adhesion strength are also possible including a homogeneous receptor distribution on the parasite surface, but will not change our general conclusions.

To complete the energetic contributions during invasion, we associate a line tension γ with the tight junction (3,4), where the parasite squeezes through the erythrocyte membrane into the nascent parasitophorous vacuole (14). The line tension may arise either from proteins within the tight junction itself, from lipid segregation next to the entering cell (48–50), from stretching of the cortical spectrin cytoskeleton underlying the erythrocyte membrane, from sharp bending of the membrane next to the tight junction, or a combination of these contributions. Either way it acts as a natural demarcation line between regions with different biophysical properties: the membrane at the site of invasion within the boundaries of the line tension and the membrane beyond (i.e., outside of the boundary of the line tension), where the spectrin cytoskeleton is expected to remain intact.

The parameters κ, c_0, σ, w, and γ together with the shape of the merozoite thus determine the energetic cost for the erythrocyte membrane deformation required for entry. These parameters are illustrated in Fig. S1 (see the Supporting Material). Electron microscopy images in Fig. S2 *A* show close contact of the merozoite and the erythrocyte membrane that motivates the adhesion energy contribution.

The absolute values for the model parameters can be translated into dimensionless parameters using the radius of a sphere with the same surface area as the parasite, a, as the basic length scale of the system, and the membrane bending rigidity κ as the energy scale. These dimensionless parameters indicated by a tilde, $\tilde{c}_0 = c_0 a^2 H_0$, $\tilde{\sigma} = \sigma a^2 / 2\kappa$, $\tilde{w} = w H_0 4\pi a^2 / 2\kappa$, and $\tilde{\gamma} = \gamma a / 2\kappa$. The average mean curvature of the merozoite can be calculated as surface integral using the archetypal merozoite defined in the next section, $H_0 = \int_{merozoite} dS\, H / \int_{merozoite} dS = 2.5/a$. The spontaneous curvature can be used to construct an effective adhesion strength, $\tilde{w}_{eff} = \tilde{w} + \tilde{c}_0$, and an effective surface tension, $\tilde{\sigma}_{eff} = \tilde{\sigma} + \tilde{c}_0^2/(aH_0)^2$, such that the phases for different values of the spontaneous curvature can be extracted using the effective parameter values.

RESULTS AND DISCUSSION

An archetypal merozoite

To calculate the contribution of erythrocyte membrane wrapping to malaria parasite invasion, we first had to develop a standardized model of a blood stage parasite. Deriving figures for such a cell from any imaging approach

is not trivial, because each naturally produces errors as a result of cryopreservation or fixation with wide associated variances (6). We have recently shown that cryo-x-ray tomography preserves physical parameters of the blood stage merozoite most accurately (6). Using this approach, we derived experimental measurements from 11 reconstructions of cryopreserved merozoites for length and width as well as estimates for mean volume and surface area. This enabled us to mathematically define an archetypal merozoite (Fig. 2, *A–B*).

Mean physical measurements were L = (1.98 ± 0.08) μm length, W = (1.40 ± 0.06) μm width, with volume and surface area averaging V_{actual} = (1.71 ± 0.15) μm^3 and A_{actual} = (8.06 ± 0.72) μm^2, respectively, where the errors are given by the standard deviations of the measurements. These dimensions give a width/length ratio of the egg-shaped merozoite as 0.71. These measurements led to a model particle that allows a mathematical description of merozoite shape with a pointed apex and rounded base (as shown in Fig. 2 *C*). This was used throughout subsequent energetic calculations. It is currently unclear whether surface convolutions observed at the macroscopic level are indicative of a native ruffled organization at the merozoite surface or an artifact of imaging. For modeling purposes, we therefore assume (conservatively) that the merozoite has a smooth surface. Conceptually, incorporation of ruffling or rippling would provide additional contact area and thus adhesion energy, but would also increase the bending-energy costs.

The egg shape of the merozoite is defined by $(x^2 + y^2 + z^2)^2 = R_a x^3 + (R_a - R_b)x(y^2 + z^2)$ with $R_a = 1$ μm and $R_b =$ 0.7 μm, which also describes the shape of a chicken egg (51). We find good agreement for surface area ($A_{idealized}$) and volume ($V_{idealized}$) of this idealized merozoite with the values measured experimentally. We use two constants, k_1 and k_2, where $A_{idealized} = k_1 L^2$ and $V_{idealized} = k_2 L^3$ to characterize the shape. For the idealized merozoite, $k_1 = 2.04$, giving $A_{idealized} = k_1 L^2 = 8.01$ μm^2. The surface area derived from rendered X-ray images of the merozoite solves k_1 as $A_{actual}/L^2 = 2.06$. For the idealized merozoite, we find $k_2 = 0.27$, giving $V_{idealized} = k_2 L^3 = 2.08$ μm^2. The volume measured from the rendered x-ray images above solves k_2 as $V_{actual}/L^3 = 0.22$. Comparison of the surface area of the idealized merozoite with the surface area of a sphere, $4 \pi a^2 = 8.01$ μm^2, defines a characteristic length scale $a = 0.8$ μm for the wrapping model described below.

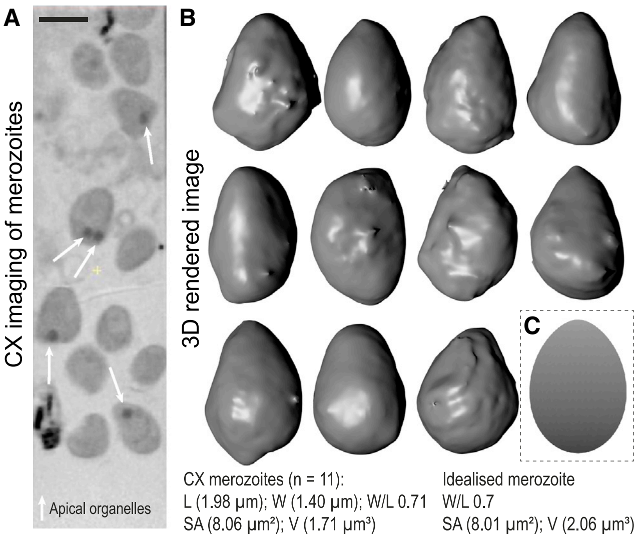

FIGURE 2 Defining an idealized archetypal merozoite. (*A*) A section through cryo-x-ray imaged free *P. falciparum* merozoites cryopreserved in a capillary. Apical secretory organelles (specifically *rhoptries*) are visible as dense spots indicated by arrows. (*B*) Isosurface rendered merozoites from (*A*). (*C*) The idealized archetypal merozoite simulated as an asymmetrical egg-shaped rigid particle. To see this figure in color, go online.

Merozoite attachment and reorientation via erythrocyte membrane wrapping

Merozoites, at egress from the infected erythrocyte, are released into the blood stream with an array of surface-bound membrane proteins (MSPs) (1). Concurrent with release, apical organelles (specifically the micronemes) commence secretion of additional classes of high-affinity binding ligands onto the surface, which diffuse toward the merozoite base (7,10,52,53). This defines a two-stage adhesive surface potential ranging from low affinity and evenly distributed at egress to high affinity, with an apical bias, before or at commencement of the invasion process beginning with reorientation.

Traditionally, merozoite reorientation has been viewed as occurring either via random rolling of the parasite or being entirely parasite driven (13) with few studies considering host-cell membrane dynamics (15,39). In the absence of directional motility (which has not been described for the free merozoite), we expect the merozoite to hit a target erythrocyte in random orientation. This primary, loosely attached state (governed by surface MSPs) involves very shallow wrapping and is clearly reversible (11,12). To reach a state of stable attachment, the energy gain due to the adhesion strength has to exceed the bending-energy cost for wrapping the erythrocyte membrane around the merozoite. Because the tight junction has not yet formed at reorientation and the membrane is not yet stretched, Eq. 2 reduces to its first and third term only (28), i.e., reorientation in our model is determined by the membrane bending rigidity and the adhesion strength only.

By comparing the bending energy and the adhesion energy at the point of contact, we find the critical adhesion strength w^* (or the dimensionless value $\tilde{w}^* = w^* a^2/(2\,\kappa)$). The bending-energy cost is proportional to the squared local mean curvature of the merozoite and, for a homogeneous adhesion strength on the merozoite surface, binding with the least curved point at the side of the merozoite is thus energetically favorable. The distribution of local adhesion

strength, which is required to induce merozoite adhesion in all orientations with equal probability, is plotted in Fig. 3. From the minimal reduced adhesion strength $\tilde{w}^* = w^* a^2/(2\,\kappa) = 5$, we can estimate a minimal adhesion strength for this stable attached binding as $w^* \approx 10^{-4}\ k_BT/nm^2$ for an archetypal merozoite with $a = 0.8\ \mu m$ and bending rigidity $\kappa = 50\ k_BT$. This value is below those of conventional receptor-ligand bonds (e.g., involved in viral invasion (54)), which could be expected for the invasion proteins known to be present on the merozoite's surface during entry. Thus, at reasonable levels of surface-protein binding to the erythrocyte membrane a minimal adhesion strength readily leads to stable attachment of the nonorientated merozoite.

To achieve a tip-first orientation of the merozoite, a gradient of the adhesion strength that favors attachment of the tip over other orientations is required. As discussed previously, such a gradient of adhesion strength from apex to base is entirely reasonable. Apical membrane antigen 1 (AMA-1), is translocated onto the merozoite surface at parasite egress (53), existing in a clear apical-basal gradient, which then freely diffuses around the merozoite periphery (52). To achieve reorientation each newly formed adhesion toward one end will require detachment at the side opposing the rolling direction. As long as the difference of the sum of adhesion and bending energy between newly formed and lost adhesion sites is negative, an energy funnel will drive merozoite rolling and reorientation. The adhesion strength at the tip has to be about nine times higher than at the side of the merozoite for it to reorient to the apex.

Evidence that inhibition of AMA-1 function disrupts merozoite reorientation directly supports its involvement in apical realignment by a natural apical-basal adhesive gradient (55). Wrapping forces and their change down an energy gradient during reorientation alone could therefore entirely explain apical reorientation without a need for parasite motor force or for host membrane buckling. Entrapment in metastable states that correspond to local minima in the energy landscape for reorientation may be overcome by additional input of energy from motor forces. Evidence for the low rates of invasion efficiency of free merozoites, however, could suggest that arrest in such energetic dead ends is a major cause for failed invasion (35).

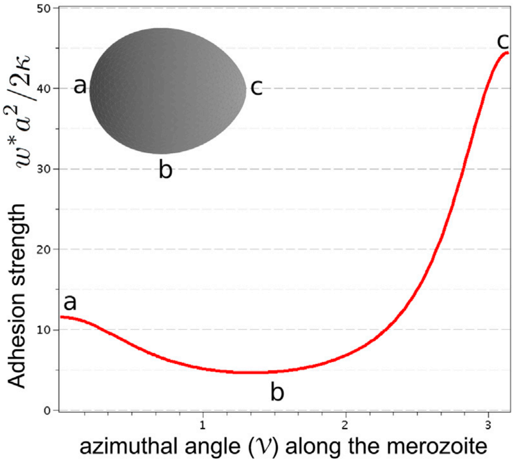

FIGURE 3 Modeling adhesive interactions between the merozoite and erythrocyte. Calculated threshold adhesion strength (using Eq. 1 with bending and adhesion terms only) $w^*a^2/(2\kappa)$ for attachment of the merozoite as a function of the azimuthal angle in a polar representation of the merozoite shape: (*a*) $v = 0$, (*b*) $0 > v > \pi$, and (*c*) $v = \pi$ correspond to the flat basal end, the side, and the apex adhered to the membrane, respectively (see Materials and Methods). To see this figure in color, go online.

Merozoite invasion via erythrocyte membrane wrapping

We next sought to determine the contribution of membrane wrapping for the actual process of invasion. True invasion, involving formation of a junction and a nascent parasitophorous vacuole, is only initiated once apical reorientation has occurred (11). At this point in time, three key cellular and molecular events occur: 1), secretion of a complex of proteins across the erythrocyte membrane that likely define two sides of the merozoite erythrocyte tight junction; 2), secretion of membrane material from parasite apical stores (namely the rhoptries); and 3), activation of a parasite actomyosin motor. Toward assessing how the contributions of membrane-wrapping might facilitate invasion, post attachment and reorientation, we calculated numerically phase diagrams of the wrapping states of the system based on the native tip-first orientation and the global minimum in the energy landscape for a given adhesion strength (Fig. 4).

In Fig. 4 *A*, the state of the system is characterized by adhesion strength w, membrane tension σ, and for a fixed line tension γ, i.e., a fixed energy cost per length for the tight junction between parasite and red cell. The value that we used for the line tension, $\gamma \approx 0.1\ k_BT/nm$ is comparable to the line tension at lipid domain boundaries (56), and might mimic a line tension due to protein aggregation. However, our model does not rely on this specific value and can be used for any value of the line tension that might be generated by one of the other mechanisms described in the Materials and Methods section. In Fig. 4 *B*, we show a phase diagram for vanishing membrane tension and the phases are plotted for various values of adhesion strength and line tension. In both phase diagrams, we find parameter regimes where the merozoite is free (non wrapped, NW), where it is partially wrapped by the erythrocyte membrane (PW I and PW II), and where it is completely wrapped (CW) (see also Fig. 1). A more detailed discussion of the phase diagrams can be found in the Supporting Material.

For small adhesion strengths, the merozoite does not attach to the erythrocyte (NW). For higher adhesion strengths, PW I, PW II, and CW states are found. From a physical point of view, adhesion strengths $5 \lesssim \tilde{w} \lesssim 15$, where partially wrapped states are found, are likely of most significance

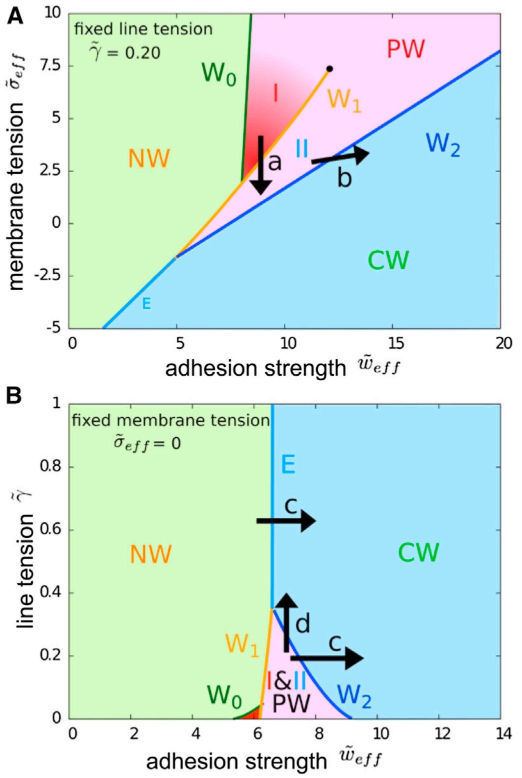

FIGURE 4 Wrapping phase diagram for fixed line tension or membrane tension. (*A*) Wrapping states of the system of a tip-first-oriented merozoite for fixed reduced line tension $\tilde{\gamma} = 0.20$ and several values of effective adhesion strength and effective membrane tension: non wrapped merozoite (NW), partially wrapped merozoite with small (PW I) and high wrapping fractions (PW II), and completely wrapped/fully invaded merozoite (CW), see Fig. 1. The transition W_0 is a continuous transition, whereas the transitions W_1, W_2, and E are associated with energy barriers. The transition W_1 ends at a critical point where the difference between PW I and PW II vanishes. The terms in Eq. 2 can be rearranged, such that the spontaneous curvature c_0 can be combined with the membrane tension and the adhesion strength to an effective membrane tension, $\tilde{\sigma}_{eff} = \tilde{\sigma} + \tilde{c}_0^2/(aH_0)^2$, and an effective adhesion strength, $\tilde{w}_{eff} = \tilde{w} + \tilde{c}_0$, respectively. The critical point is indicated by a black point (•). (*B*) Wrapping states of the system of a tip-first-oriented merozoite for vanishing effective membrane tension, $\tilde{\sigma}_{eff} = 0$, and several values of effective adhesion strength and line tension. The notation is analogous to *A*. Both phase diagrams with additional wrapping spinodals that indicate the values of the adhesion strength beyond which the energy barrier vanishes and spontaneous wrapping occurs, are shown in Fig. S3. However, the energy barriers may also be crossed by other mechanisms: arrow ***a*** indicates the effect of unstructured membrane secreted by the merozoite (Fig. S2 *B*), arrow ***b*** shows the effect of favorable spontaneous curvature, arrow ***c*** the effect of increased adhesion strength, and arrow ***d*** the effect of increased line tension. To see this figure in color, go online.

with regard to invasion energetics. The values of w for the transition to wrapped states are given by the phase boundaries between the NW regime to PW regimes (W_0 and the part of W_1 for small surface and line tensions) and the boundary between NW and CW regimes (E). For low surface tensions ($\tilde{\sigma} < 7.5$) there is an energy barrier between PW states with a small and high wrapping fractions, whereas for a large enough surface tension ($\tilde{\sigma} \gtrsim 7.5$), the energy barrier between PW I and PW II disappears and the wrapping fraction increases continuously with the adhesion strength. Large adhesion strengths allow immediate complete wrapping and erythrocyte entry, but might also be associated with unspecific binding to other membranes and problems associated with membrane surface-coat shedding. Thus, lower affinity interactions seem to be favorable. Fig. 4 shows that a minimal value $\tilde{w} \approx$ 5–7 is large enough to generate a stable, PW state. Successful invasion requires an end state in the CW region of the phase diagram. However, if the energy barrier of the transition to the CW state is high but invasion is assisted by additional forces, such as motor forces (see below), successful invasion might occur already for smaller adhesion strengths.

Fig. 5 shows the critical wrapping fractions of the merozoite surface area between which the W_1 and W_2 transitions (shown in Fig. 4 *A*) occur as a function of the reduced adhesion strength for a reduced line tension $\tilde{\gamma} \approx 0.2$. In the PW region I, tip-wrapped states (as experimentally observed in early stages of invasion) occur with <20% of the merozoite surface area in contact with the erythrocyte. States with a higher wrapping fraction of the merozoite up to 90% are found in the PW region II. In Fig. 5 the hatched regions correspond to unstable states that form the energy barriers associated with the discontinuous phase transitions. The unstable region between the PW states vanishes for adhesion strengths larger than $\tilde{w} = 12$, which corresponds to the critical point in Fig. 4 *A*.

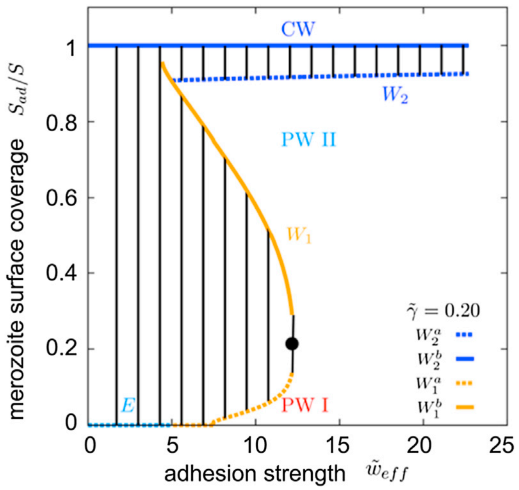

FIGURE 5 Merozoite surface coverage for different adhesion strengths. Merozoite surface coverage for different adhesion strengths and reduced line tension $\tilde{\gamma} = 0.20$. W_1, W_2, and E, are the phase boundaries and lines demarking equal energy of free and CW states, see Fig. 4 *A*. The critical point is indicated by a black point (•). The wrapping fractions where the system jumps between discontinuous transitions are indicated using the dotted or the solid lines, corresponding states are connected by tie-lines. W_1^a and W_2^a indicate lower, whereas W_1^b and W_2^b higher wrapped fractions of the merozoite surface area for the W_1 and W_2 transitions, the two phase regions in the phase diagram indicated by the black tie-lines are unstable and correspond to the energy barriers. To see this figure in color, go online.

The line tension, at which the erythrocyte membrane detaches from the merozoite, is a key determinant for the stability of PW states, both hindering the entry of the parasite for early stages and favoring wrapping at later stages (Fig. 4 *B*, *arrow d*). This contact line, seen as an electron-dense zone in electron micrographs (3,6), must be stretched at the onset of entry and will contract after the point of maximal diameter of the merozoite has passed. In the phase diagram in Fig. 4 *B*, invasion is considered with respect to wrapping states across values of line tension and adhesion strength, while keeping the adhesion strength fixed. For small values of the line tension, PW states with high wrapping fraction are found, whereas for large values of the line tension (larger than $\tilde{\gamma} \approx 0.35$) the transition occurs directly from the free, NW state to the CW, invaded state. In this way, a robust line tension helps to facilitate complete invasion and avoid PW states with high wrapping fraction. Thus, from a biological point of view, there are clear adaptive advantages associated with formation of a demarking tension between the parasite and host cell. This may in part explain the origin of the tight junction as a strategy for differentiating between membrane regions and contributing to reducing the energetic requirements for reaching an invaded state rather than, necessarily, only as a point of motor traction (16).

During the invasion process, experimental evidence clearly shows both secretion of unstructured membrane by the merozoite (discharged from the rhoptry organelles) and gross changes in the membrane curvature of the erythrocyte (6,7,13,57) (Fig. S2 *B*). The effect of such events on the status of invasion can be directly interpreted in light of the phase diagram in Fig. 4. When the lipid bilayer area of the erythrocyte is increased by additional unstructured membrane from the parasite, the tension of the membrane that wraps the merozoite is lowered considerably. This corresponds to a move in the wrapping diagram from a PW to a CW state (Fig. 4 *A*, *arrow a*). In addition to extra membrane area being provided, the unstructured nature of this membrane as well as any detachment or reorganization of the spectrin cytoskeleton from the bilayer (19,39) can change the spontaneous curvature of the membrane to a value that is more favorable for wrapping (Fig. 4 *A*, *arrow b*). Mathematically, the spontaneous curvature can be taken into account using the effective adhesion strength and the effective surface tension, otherwise leaving the wrapping phase diagrams unchanged (see Materials and Methods). These results point to specific benefits that would arise from local disruption of the spectrin network, either directly or indirectly, by the merozoite, something that has been observed empirically by electron microscopy of invading parasites (58).

Merozoite invasion supported by motor activity

Having explored the role of adhesive forces (from parasite invasion adhesins), bending rigidity, membrane tension, line tension, and spontaneous curvature of the erythrocyte membrane to membrane wrapping states, we next sought to estimate the degree of active forces required from the parasite to overcome energy barriers and facilitate transitions to completed invasion. The current model for the source of parasite active motor force posits that an anchored myosin motor inside the parasite cell (directly tethered to a cytoskeletal compartment within the cell pellicle) transmits force directly through a short polymerized actin filament, which itself is linked to the surface-bound adhesin. The binding of this surface adhesin to a red cell receptor and their combined passage towards the base of the merozoite length (through the fluid plasma membrane) is then thought to generate a rearward force driving the parasite forward (Fig. 6, *A* and *B*) (59).

Two energetic events could be envisaged to require the force generated by this actomyosin motor: breaking or moving aside of the erythrocyte cytoskeleton at the site of invasion (to allow entry) and overcoming wrapping energy barriers.

At the specific site of entry, we assume that the cytoskeleton of the erythrocyte gets disassembled, which is experimentally supported by evidence that there is an erythrocytic ATP requirement for invasion (60,61) and the dependency of cytoskeletal reorganization on ATP (43,62). This implies that, at the onset of true invasion (postattachment), the merozoite is wrapped by a membrane without an explicit contribution of the shear modulus. For local disassembly of the cytoskeleton, a stretching force would be required that may be contributed by motor activity. A rough estimate shows that for the cross-sectional area of the merozoite (~1 μm^2 (6)), a few hundred spectrin bonds would have to be broken/segregated to accommodate an entering merozoite (for a spectrin bond length of ~60–100 nm, the average length between the protein complexes that bind the ends of the spectrin filaments to the lipid bilayer membrane (25)).

The second, and more obvious energetic barrier requiring parasite motor force in our model is clear from the stable states depicted in Fig. 4. For intermediate adhesion strengths, energy barriers (see the Supporting Material) separate the PW states internally (W_1) and the completely invaded state from the PW states (W_2). The energy barriers between NW and CW states decrease with increasing adhesion strength (32) but increase with increasing line tension. Spontaneous invasion from adhesion alone could only occur with unreasonably large adhesion strengths combined with a small line and surface tensions. Therefore, it is far more likely that application of force by the merozoite itself facilitates the transitions between NW, PW, and CW states. Estimates of the required motor activity to wrap the merozoite can be derived from the force calculated for merozoite invasion without adhesion energy (Fig. 6 *C*). The maximal force F_z (besides a very small wrapping fraction below 5%) is of the order of $20\ \kappa/a \approx 1\ k_BT/nm$, or ~5 pN. Typical estimates of the force related to a single, motility-dependent adhesion site for a *Plasmodium* preerythrocytic parasite on a

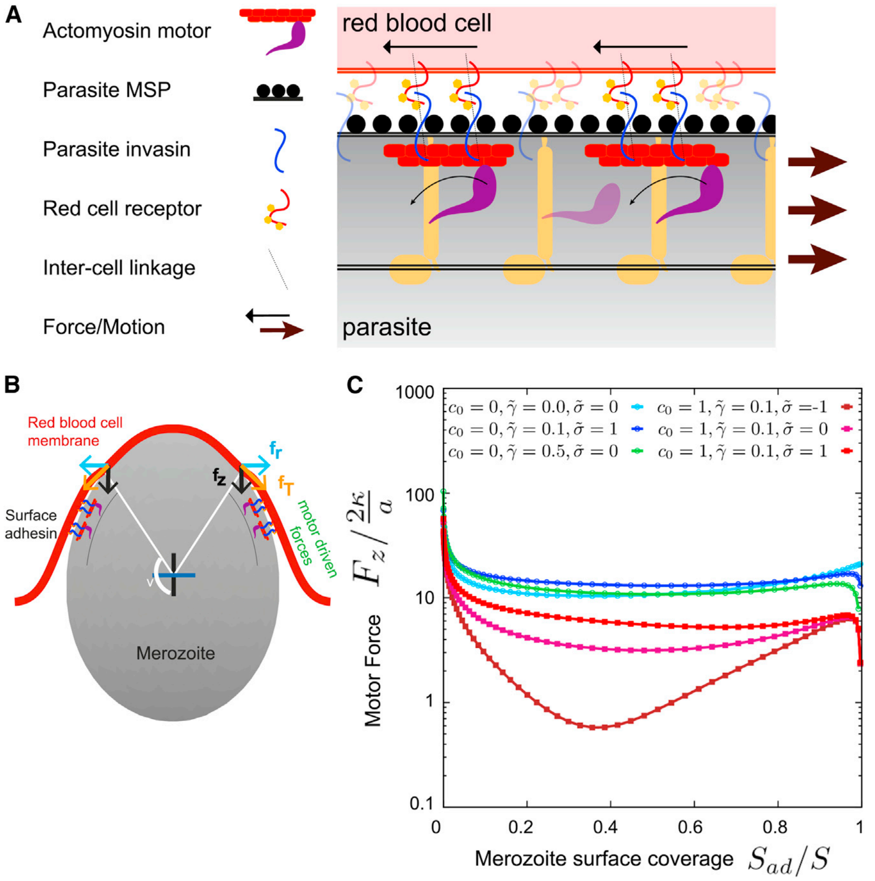

FIGURE 6 The contribution of motor driving forces in merozoite invasion. (*A*) The current model for the topology of the parasite actomyosin motor and its linkage with the red blood cell surface through secreted invasins. (*B*) Actomyosin force supports merozoite invasion. f_T is the force acting tangentially along the membrane-cortex surface as it wraps along the particle, whereas the f_z is the component of this tangential force along the *z* axis whose role is to inject the particle into the membrane while the component f_r is balanced by an equal magnitude force acting along the other side of the membrane. (*C*) Estimate for the motor-driven forces required to push a nonadhesive merozoite into the erythrocyte membrane to achieve complete invasion. $F_Z = 2\pi r f_z$, is the estimate for the total invasive force acting along the symmetry axis required to inject the merozoite as a function of the fraction of the merozoite surface covered by erythrocyte membrane; *r* is the radius of the rim where the merozoite detaches from the membrane. To see this figure in color, go online.

glass substrate (though not necessarily a single motor) are ~6.5 pN (63). Therefore, a small number of adhesion sites in the context of an invasion event could be easily sufficient to overcome the energy barriers required for entry. *T. gondii* myosin A, considered the direct ortholog of that involved in merozoite invasion (64), has a step size of 5.2 nm (65). Although its stall force is not known, most myosin types generate forces in the order of 0.5 to 5 pN per motor molecule (66,67). Given that myosin A is only single headed and cannot be processive, and assuming a small duty ratio of 5% (approximately that for muscle myosin (68)), at each instant between 2 and 10 motors should be bound. Using a stochastic attachment model and assuming 5 motors are bound, invasion would require ~5/0.05 = 100 motors (lower and upper limits being 40 to 200 motors) to perform without failure under these conditions.

As shown in Fig. 6 *C*, the required force for invasion can be reduced by orders of magnitude if factors such as favorable spontaneous curvature of the erythrocyte membrane or expulsion of unstructured membrane accompany invasion. There is strong evidence for both (see above), which suggests that the energetic contribution of the motor estimated previously could be much lower. Irrespective of the alternative contributions of parasite adhesion or membrane remodeling, the need for an active directional motor force, such as that generated by the parasite actomyosin machinery, cannot be eliminated: due to energy barriers between stable membrane-wrapped states (see the Supporting Material), the motor likely plays an essential role for achieving robust invasion. In this way, our modeling is consistent with experimental evidence suggesting that actomyosin motor activity is essential for merozoite invasion (16,17,64). Residual invasion following complete motor inactivity in the related apicomplexan *T. gondii* might suggest alternative host-cell processes that are not possible in the erythrocyte (such as phagocytosis) or redundant invasion machinery in the parasite that is currently unaccounted for (22). What is clear, however, is that at its most energy efficient, theoretical predictions for erythrocyte invasion envision a balance between passive host cell-dependent processes (nonetheless stimulated and controlled by the parasite) and those that are parasite-energy dependent. This rejects the perceived dogma wherein merozoite invasion is entirely parasite driven.

CONCLUSIONS: A COMPLETE BIOPHYSICAL MODEL FOR INVASION INCORPORATING MEMBRANE WRAPPING

By integrating basic parasitological observations of merozoite maturation and surface protein biology with biophysical consideration of membrane wrapping, we can now propose a complete mechanistic model of invasion (Fig. 7).

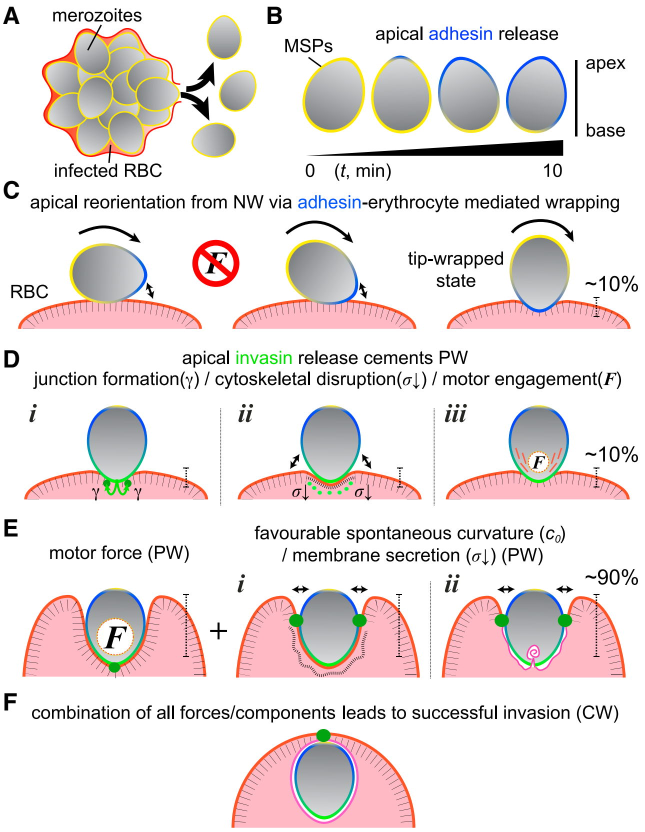

FIGURE 7 Schematic for biophysical interactions between the *Plasmodium* merozoite and the human erythrocyte. A complete biophysical model for merozoite invasion of the erythrocyte from release (*A* and *B*), to attachment and reorientation facilitating a stable, tip-wrapped state (*C*), to PW states (*D* and *E*), and full invasion/CW states (*F*). See main text for details. To see this figure in color, go online.

At the outset, it is clear that the early stages of invasion are setup at merozoite egress from the infected erythrocyte (10,53,69). Daughter merozoites are liberated into the blood stream (Fig. 7 *A*) with a surface studded evenly with MSPs, which provide low-strength reversible (and nonorientated) attachment to a target erythrocyte (1) (Fig. 7 *B*). At egress, a second, variable population of adhesive proteins (which we term adhesins (8)) are released apically from secretory micronemes (Fig. 7 *B*). Their diffusion through the merozoite plasma membrane sets up a transient adhesive gradient from apex to base on its surface (Fig. 7 *B*). This gradient is biologically dependent on many factors, not least the timing of release (53), and may compensate the increased bending-energy cost at the apex required for merozoite reorientation. The initial apical gradient transitions the merozoite from a reversible (MSP determined) to an irreversible (adhesin determined) attached state on the target erythrocyte surface that can be sufficient alone to reorientate the merozoite without parasite energy-dependent forces (Fig. 7 *C*). Of note, mature merozoites with complete surface release of adhesins (and homogeneity through the plasma membrane at times tending toward ~10 min, Fig. 7 *B*) will be unable to reorientate by wrapping forces alone, possibly explaining their short invasion half-life (35). Once reorientation has occurred, the merozoite typically exists in a stable, tip-wrapped/PW state with <10% of the parasite wrapped (Fig. 7 *C* and Figs. 4 and 5, PW I).

An ~10% PW state (Fig. 7 *C*, *right panel*) is also found in our wrapping energy calculations (Figs. 4 and 5, PW I) and in the biological system likely coincides with (or is directly preceded by) apical release of a third population of invasion-specific adhesive proteins, which we term invasins (8) (*green*) to distinguish them from adhesins. These are associated with either a second population of micronemes or the rhoptry organelles (8,70) (Fig. 7 *D*). Some of these are secreted inside the target erythrocyte (7), with others staying with the merozoite surface (8). Combined, the rhoptries and the proteins released facilitate four key processes (Fig. 7 *D* and Fig. 4, *arrows*): i), establishment of the merozoite-erythrocyte tight junction (*dark green*), which in our model acts as line tension (γ); ii), a (still to be determined) class of proteins, predicted to be secreted inside the erythrocyte, which may facilitate disruption of the underlying erythrocyte cytoskeleton (*thick to thin dashed line*) (19,71) leading to a more favorable spontaneous curvature (c_0); iii), secretion of membrane from the merozoite's apical stores (7,57) lowering the tension of the erythrocyte membrane (σ) facilitating further wrapping (Fig. 7 *E*, *right panel*, and Fig. 4, *arrow a*), which has a favorable spontaneous curvature and reduces surface tension; and iv) a final class of proteins, released onto the surface of the merozoite, which engage with the actomyosin motor inside the merozoite permitting force generation (***F***) (72). We suggest that each of these factors helps the merozoite to overcome energy barriers associated with transitions between low and high PW states (Fig. 4, Fig. 5, and Fig. 7, *D* and *E*). Motor force allows the merozoite to then cross the remaining energy barriers and to achieve invasion up to ~90% wrapping. At this critical juncture in invasion (Fig. 7 *E*, Fig.S3, and Fig. S5, PW II), the merozoite will either jump to a CW/invaded state (Fig. 7 *F*) or invasion will fail. In reality, these scenarios likely coexist and are continuations of the previous stages of invasion. They are also entirely reasonable given known experimental observations (e.g., (57,71), Fig. S2 *B*). The successfully invaded parasite now lies within a vacuole inside the target erythrocyte (14). At this stage, the vacuole will need to be sealed and erythrocyte cytoskeleton reformed.

Two core conclusions can be drawn from this model. First, that reorientation to irreversible attachment can be simply viewed as a parasite energy-independent, shape/adhesin-dependent wrapping process. Second, that membrane wrapping during merozoite invasion combined with other biophysical considerations can account for the major energetic requirements of invasion. Successful invasion requires traversal across energy barriers (associated with the discontinuous transitions), which are likely achieved

via actomyosin motor contributions. However, what is striking is that our calculations for membrane wrapping together with biological evidence suggest mechanisms that make parasite entry into the erythrocyte energetically more favorable compared to a model that is parasite motor-driven alone. Nonmotor contributions such as cytoskeletal remodeling and the line tension from the junction thus contribute to invasion energetics in ways that have perhaps not been appreciated. It is worth stressing that, irrespective of wrapping models, motor force does still appear to be a constant requirement (17,64). Our work demonstrates that even when membrane-wrapped states are stable, the essential role of the motor likely lies in overcoming energy barriers between the PW and CW states.

Clearly, it is now paramount to actually measure the forces experienced by the merozoite, and assess the contributions from membrane-wrapping and myosin motors. In addition, direct assessment of the membrane contributions from the parasite apex to the nascent vacuole (14) and the search for factors that modulate the erythrocyte cytoskeleton either directly or indirectly to facilitate invasion (19) also become important. This latter point is particularly worth highlighting. A parasite-induced role for the host cell is clearly documented if one looks beyond *Toxoplasma* and *Plasmodium* spp. to other apicomplexan parasites (73–75). Could the erythrocyte be stimulated to contribute in a similar fashion to invasion? The erythrocyte membrane and cytoskeleton play a particularly active role during erythroblast enucleation, which involves extensive cytoskeletal remodeling that helps expel the nuclear compartment (76,77). Given the presence of such a machinery combined with evidence that the mature erythrocyte cytoskeleton and membrane exist in a dynamic cycling state (43,78) it is certainly conceivable that the parasite might stimulate innate active processes within the erythrocyte to further reduce the energy cost for entry.

In summary, our results point to an evolutionarily balanced consideration of merozoite invasion, strongly favoring a model by which passive processes, such as adhesion-driven wrapping, and active parasite-induced processes, such as erythrocyte cytoskeleton remodeling and membrane injection (and of course motor contribution), combine to maximize invasion efficiency. Strategies targeting either of these facets of invasion, or a combination of the two, may therefore be important as we strive for more effective antimalarial therapeutics.

SUPPORTING MATERIAL

Four figures, supporting data, some details of the theoretical analysis, a discussion of discontinuous transitions in the phase diagram, and reference (79) are available at http://www.biophysj.org/biophysj/supplemental/S0006-3495(14)00558-X.

This work was the result of a “*Meeting of Minds*” symposium held at the Institut Pasteur, Paris and we are grateful to the Mairie de Paris, OzEMalaR (the Australia/Europe Malaria Research Cooperative) and the Institut Pasteur, Paris for facilitating the meeting. The authors thank Robert Menard for critical input into the study and Carolyn Larabell, Mark A. Le Gros (University of California, San Francisco), and Christian Knoechel (Lawrence Berkeley National Laboratory) for assistance and advice with x-ray tomography undertaken at the National Center for X-ray Tomography, Lawrence Berkeley National Laboratory.

X-ray tomography work was supported by grants from the National Center for Research Resources (5P41RR019664-08) and the National Institute of General Medical Sciences (8 P41 GM103445-08) from the National Institutes of Health. Direct funding to support parasitological work was from the NHMRC (Project Grant APP1047085, JB) and, for theoretical work, from the EU FP7 NMP collaborative project PreNanoTox (Project Grant 309666, GG). S.D. acknowledges support by the International Helmholtz Research School of Biophysics and Soft Matter (IHRS BioSoft). E.S.Z. is supported by an Australian Postgraduate Award (APA); N.S.G. thanks the Mayent-Rothschild Visiting Professor Grant at the Institut Curie for funding; T.J.S. is supported by a Wellcome Trust project grant (No. 094277). A.M.T. is supported by an NHSBT R&D grant and the Wellcome Trust; T.B. acknowledges support from the Agence Nationale de Recherche (JCJC SVSE 5-2011); J.B. was supported by a Future Fellowship from the Australian Research Council, ARC (FT100100112) and is currently supported by a New Investigator Award from the Wellcome Trust (100993/Z/13/Z). Author contributions: S.D., T.A., N.G., T.J.S., E.H., E.S.Z., D.T.R., A.M.T., T.B., J.B., and G.G. worked closely together to design, perform, and interpret experiments; S.D., T.A., N.G., T.B., J.B., and G.G. wrote the paper.

REFERENCES

1. Cowman, A. F., D. Berry, and J. Baum. 2012. The cellular and molecular basis for malaria parasite invasion of the human red blood cell. *J. Cell Biol.* 198:961–971.
2. Baum, J., T.-W. Gilberger, …, M. Meissner. 2008. Host-cell invasion by malaria parasites: insights from *Plasmodium* and *Toxoplasma*. *Trends Parasitol.* 24:557–563.
3. Aikawa, M., L. H. Miller, …, J. Rabbege. 1978. Erythrocyte entry by malarial parasites. A moving junction between erythrocyte and parasite. *J. Cell Biol.* 77:72–82.
4. Bannister, L. H., G. A. Butcher, …, G. H. Mitchell. 1975. Structure and invasive behavior of *Plasmodium knowlesi* merozoites in vitro. *Parasitology.* 71:483–491.
5. Ladda, R., M. Aikawa, and H. Sprinz. 1969. Penetration of erythrocytes by merozoites of mammalian and avian malarial parasites. *J. Parasitol.* 55:633–644.
6. Hanssen, E., C. Dekiwadia, …, S. A. Ralph. 2013. Electron tomography of *Plasmodium falciparum* merozoites reveals core cellular events that underpin erythrocyte invasion. *Cell. Microbiol.* 15:1457–1472.
7. Riglar, D. T., D. Richard, …, J. Baum. 2011. Super-resolution dissection of coordinated events during malaria parasite invasion of the human erythrocyte. *Cell Host Microbe.* 9:9–20.
8. Zuccala, E. S., A. M. Gout, …, J. Baum. 2012. Subcompartmentalization of proteins in the rhoptries correlates with ordered events of erythrocyte invasion by the blood stage malaria parasite. *PLoS ONE.* 7:e46160.
9. Srinivasan, P., W. L. Beatty, …, L. H. Miller. 2011. Binding of *Plasmodium* merozoite proteins RON2 and AMA1 triggers commitment to invasion. *Proc. Natl. Acad. Sci. USA.* 108:13275–13280.
10. Singh, S., M. M. Alam, …, C. E. Chitnis. 2010. Distinct external signals trigger sequential release of apical organelles during erythrocyte invasion by malaria parasites. *PLoS Pathog.* 6:e1000746.
11. Dvorak, J. A., L. H. Miller, …, T. Shiroishi. 1975. Invasion of erythrocytes by malaria merozoites. *Science.* 187:748–750.

12. Gilson, P. R., and B. S. Crabb. 2009. Morphology and kinetics of the three distinct phases of red blood cell invasion by *Plasmodium falciparum* merozoites. *Int. J. Parasitol.* 39:91–96.

13. Mitchell, G. H., and L. H. Bannister. 1988. Malaria parasite invasion: interactions with the red cell membrane. *Crit. Rev. Oncol. Hematol.* 8:225–310.

14. Lingelbach, K., and K. A. Joiner. 1998. The parasitophorous vacuole membrane surrounding *Plasmodium* and *Toxoplasma*: an unusual compartment in infected cells. *J. Cell Sci.* 111:1467–1475.

15. Lew, V. L., and T. Tiffert. 2007. Is invasion efficiency in malaria controlled by pre-invasion events? *Trends Parasitol.* 23:481–484.

16. Angrisano, F., D. T. Riglar, …, J. Baum. 2012. Spatial localization of actin filaments across developmental stages of the malaria parasite. *PLoS ONE.* 7:e32188.

17. Miller, L. H., M. Aikawa, …, T. Shiroishi. 1979. Interaction between cytochalasin B-treated malarial parasites and erythrocytes. Attachment and junction formation. *J. Exp. Med.* 149:172–184.

18. Sibley, L. D. 2004. Intracellular parasite invasion strategies. *Science.* 304:248–253.

19. Zuccala, E. S., and J. Baum. 2011. Cytoskeletal and membrane remodelling during malaria parasite invasion of the human erythrocyte. *Br. J. Haematol.* 154:680–689.

20. Dobrowolski, J. M., and L. D. Sibley. 1996. *Toxoplasma* invasion of mammalian cells is powered by the actin cytoskeleton of the parasite. *Cell.* 84:933–939.

21. Gonzalez, V., A. Combe, …, I. Tardieux. 2009. Host cell entry by apicomplexa parasites requires actin polymerization in the host cell. *Cell Host Microbe.* 5:259–272.

22. Andenmatten, N., S. Egarter, …, M. Meissner. 2013. Conditional genome engineering in *Toxoplasma gondii* uncovers alternative invasion mechanisms. *Nat. Methods.* 10:125–127.

23. Huang, J., M. Juszkiewicz, …, T. P. Russell. 2007. Capillary wrinkling of floating thin polymer films. *Science.* 317:650–653.

24. Vliegenthart, G. A., and G. Gompper. 2006. Forced crumpling of self-avoiding elastic sheets. *Nat. Mater.* 5:216–221.

25. Ursitti, J. A., D. W. Pumplin, …, R. J. Bloch. 1991. Ultrastructure of the human erythrocyte cytoskeleton and its attachment to the membrane. *Cell Motil. Cytoskeleton.* 19:227–243.

26. Auth, T., S. A. Safran, and N. S. Gov. 2007. Fluctuations of coupled fluid and solid membranes with application to red blood cells. *Phys. Rev. E Stat. Nonlin. Soft Matter Phys.* 76:051910.

27. Lipowsky, R., and H. G. Döbereiner. 1998. Vesicles in contact with nanoparticles and colloids. *Europhys. Lett.* 43:219–225.

28. Deserno, M. 2004. Elastic deformation of a fluid membrane upon colloid binding. *Phys. Rev. E Stat. Nonlin. Soft Matter Phys.* 69:031903.

29. Tollis, S., A. E. Dart, …, R. G. Endres. 2010. The zipper mechanism in phagocytosis: energetic requirements and variability in phagocytic cup shape. *BMC Syst. Biol.* 4:149.

30. Bahrami, A. H., R. Lipowsky, and T. R. Weikl. 2012. Tubulation and aggregation of spherical nanoparticles adsorbed on vesicles. *Phys. Rev. Lett.* 109:188102.

31. Šarić, A., and A. Cacciuto. 2012. Fluid membranes can drive linear aggregation of adsorbed spherical nanoparticles. *Phys. Rev. Lett.* 108:118101.

32. Dasgupta, S., T. Auth, and G. Gompper. 2013. Wrapping of ellipsoidal nano-particles by fluid membranes. *Soft Matter.* 9:5473–5482.

33. Bahrami, A. H. 2013. Orientational changes and impaired internalization of ellipsoidal nanoparticles by vesicle membranes. *Soft Matter.* 9:8642–8646.

34. Dasgupta, S., T. Auth, and G. Gompper. 2014. Shape and orientation matter for the cellular uptake of nonspherical particles. *Nano Lett.* 14:687–693.

35. Boyle, M. J., D. W. Wilson, …, J. G. Beeson. 2010. Isolation of viable *Plasmodium falciparum* merozoites to define erythrocyte invasion events and advance vaccine and drug development. *Proc. Natl. Acad. Sci. USA.* 107:14378–14383.

36. Lipowsky, R. 1992. Budding of membranes induced by intramembrane domains. *J. Phys. II France.* 2:1825–1840.

37. Helfrich, W. 1973. Elastic properties of lipid bilayers: theory and possible experiments. *Z. Naturforsch. C.* 28:693–703.

38. Gray, A., and E. Abbena. 2006. Modern Differential Geometry of Curves and Surfaces with Mathematica. CRC Press, Boca Raton, FL.

39. Kabaso, D., R. Shlomovitz, …, N. S. Gov. 2010. Curling and local shape changes of red blood cell membranes driven by cytoskeletal reorganization. *Biophys. J.* 99:808–816.

40. Auth, T., S. A. Safran, and N. S. Gov. 2007. Filament networks attached to membranes: cytoskeletal pressure and local bilayer deformation. *New J. Phys.* 9:430.

41. Seifert, U. 1995. The concept of effective tension for fluctuating vesicles. *Z. Phys. B Condens. Matter.* 97:299–309.

42. Farago, O., and P. Pincus. 2004. Statistical mechanics of bilayer membrane with a fixed projected area. *J. Chem. Phys.* 120:2934–2950.

43. Gov, N. S., and S. A. Safran. 2005. Red blood cell membrane fluctuations and shape controlled by ATP-induced cytoskeletal defects. *Biophys. J.* 88:1859–1874.

44. Tian, A., and T. Baumgart. 2009. Sorting of lipids and proteins in membrane curvature gradients. *Biophys. J.* 96:2676–2688.

45. Veksler, A., and N. S. Gov. 2007. Phase transitions of the coupled membrane-cytoskeleton modify cellular shape. *Biophys. J.* 93:3798–3810.

46. Auth, T., and G. Gompper. 2009. Budding and vesiculation induced by conical membrane inclusions. *Phys. Rev. E Stat. Nonlin. Soft Matter Phys.* 80:031901.

47. Sorre, B., A. Callan-Jones, …, P. Bassereau. 2009. Curvature-driven lipid sorting needs proximity to a demixing point and is aided by proteins. *Proc. Natl. Acad. Sci. USA.* 106:5622–5626.

48. Heberle, F. A., J. Wu, …, G. W. Feigenson. 2010. Comparison of three ternary lipid bilayer mixtures: FRET and ESR reveal nanodomains. *Biophys. J.* 99:3309–3318.

49. Schäfer, L. V., and S. J. Marrink. 2010. Partitioning of lipids at domain boundaries in model membranes. *Biophys. J.* 99:L91–L93.

50. Hutchison, J. B., R. M. Weis, and A. D. Dinsmore. 2012. Change of line tension in phase-separated vesicles upon protein binding. *Langmuir.* 28:5176–5181.

51. Yamamoto, N. 2007. We Find an Equation of Egg Shaped Curves. http://www.geocities.jp/nyjp07/Egg/index_egg_E.html. Accessed March 29, 2007.

52. Treeck, M., S. Zacherl, …, T. W. Gilberger. 2009. Functional analysis of the leading malaria vaccine candidate AMA-1 reveals an essential role for the cytoplasmic domain in the invasion process. *PLoS Pathog.* 5:e1000322.

53. Collins, C. R., F. Hackett, …, M. J. Blackman. 2013. Malaria parasite cGMP-dependent protein kinase regulates blood stage merozoite secretory organelle discharge and egress. *PLoS Pathog.* 9:e1003344.

54. Sun, S. X., and D. Wirtz. 2006. Mechanics of enveloped virus entry into host cells. *Biophys. J.* 90:L10–L12.

55. Mitchell, G. H., A. W. Thomas, …, L. H. Bannister. 2004. Apical membrane antigen 1, a major malaria vaccine candidate, mediates the close attachment of invasive merozoites to host red blood cells. *Infect. Immun.* 72:154–158.

56. García-Sáez, A. J., S. Chiantia, and P. Schwille. 2007. Effect of line tension on the lateral organization of lipid membranes. *J. Biol. Chem.* 282:33537–33544.

57. Bannister, L. H., G. H. Mitchell, …, E. D. Dennis. 1986. Lamellar membranes associated with rhoptries in erythrocytic merozoites of *Plasmodium knowlesi*: a clue to the mechanism of invasion. *Parasitology.* 92:291–303.

58. Aikawa, M., L. H. Miller, …, N. Epstein. 1981. Freeze-fracture study on the erythrocyte membrane during malarial parasite invasion. *J. Cell Biol.* 91:55–62.

59. Baum, J., A. T. Papenfuss, …, A. F. Cowman. 2006. Regulation of apicomplexan actin-based motility. *Nat. Rev. Microbiol.* 4:621–628.

60. Ayi, K., W. C. Liles, …, K. C. Kain. 2009. Adenosine triphosphate depletion of erythrocytes simulates the phenotype associated with pyruvate kinase deficiency and confers protection against *Plasmodium falciparum* in vitro. *J. Infect. Dis.* 200:1289–1299.

61. Olson, J. A., and A. Kilejian. 1982. Involvement of spectrin and ATP in infection of resealed erythrocyte ghosts by the human malarial parasite, *Plasmodium falciparum. J. Cell Biol.* 95:757–762.

62. Park, Y., C. A. Best, …, M. S. Feld. 2010. Metabolic remodeling of the human red blood cell membrane. *Proc. Natl. Acad. Sci. USA.* 107:1289–1294.

63. Hegge, S., K. Uhrig, …, F. Frischknecht. 2012. Direct manipulation of malaria parasites with optical tweezers reveals distinct functions of *Plasmodium* surface proteins. *ACS Nano.* 6:4648–4662.

64. Pinder, J. C., R. E. Fowler, …, W. B. Gratzer. 1998. Actomyosin motor in the merozoite of the malaria parasite, *Plasmodium falciparum*: implications for red cell invasion. *J. Cell Sci.* 111:1831–1839.

65. Herm-Götz, A., S. Weiss, …, D. Soldati. 2002. *Toxoplasma gondii* myosin A and its light chain: a fast, single-headed, plus-end-directed motor. *EMBO J.* 21:2149–2158.

66. Finer, J. T., R. M. Simmons, and J. A. Spudich. 1994. Single myosin molecule mechanics: piconewton forces and nanometre steps. *Nature.* 368:113–119.

67. Tyska, M. J., D. E. Dupuis, …, S. Lowey. 1999. Two heads of myosin are better than one for generating force and motion. *Proc. Natl. Acad. Sci. USA.* 96:4402–4407.

68. Harris, D. E., and D. M. Warshaw. 1993. Smooth and skeletal muscle myosin both exhibit low duty cycles at zero load in vitro. *J. Biol. Chem.* 268:14764–14768.

69. Abkarian, M., G. Massiera, …, C. Braun-Breton. 2011. A novel mechanism for egress of malarial parasites from red blood cells. *Blood.* 117:4118–4124.

70. Kremer, K., D. Kamin, …, M. Meissner. 2013. An overexpression screen of *Toxoplasma gondii* Rab-GTPases reveals distinct transport routes to the micronemes. *PLoS Pathog.* 9:e1003213.

71. Rangachari, K., A. Dluzewski, …, W. B. Gratzer. 1986. Control of malarial invasion by phosphorylation of the host cell membrane cytoskeleton. *Nature.* 324:364–365.

72. Baum, J., D. Richard, …, A. F. Cowman. 2006. A conserved molecular motor drives cell invasion and gliding motility across malaria life cycle stages and other apicomplexan parasites. *J. Biol. Chem.* 281:5197–5208.

73. Elliott, D. A., D. J. Coleman, …, D. P. Clark. 2001. *Cryptosporidium parvum* infection requires host cell actin polymerization. *Infect. Immun.* 69:5940–5942.

74. Lumb, R., K. Smith, …, J. A. Lanser. 1988. Ultrastructure of the attachment of *Cryptosporidium* sporozoites to tissue culture cells. *Parasitol. Res.* 74:531–536.

75. Shaw, M. K. 2003. Cell invasion by *Theileria* sporozoites. *Trends Parasitol.* 19:2–6.

76. Lee, J. C., J. A. Gimm, …, J. A. Chasis. 2004. Mechanism of protein sorting during erythroblast enucleation: role of cytoskeletal connectivity. *Blood.* 103:1912–1919.

77. Konstantinidis, G., A. Moustakas, and C. Stournaras. 2011. Regulation of myosin light chain function by BMP signaling controls actin cytoskeleton remodeling. *Cell. Physiol. Biochem.* 28:1031–1044.

78. Betz, T., M. Lenz, …, C. Sykes. 2009. ATP-dependent mechanics of red blood cells. *Proc. Natl. Acad. Sci. USA.* 106:15320–15325.

79. Elsa, A., S. Salamon, and A. Gray. 2006. Modern Differential Geometry of Curves and Surfaces with Mathematica. CRC Press, Boca Raton, FL.

Supporting Material

Membrane-wrapping contributions to malaria parasite invasion of the human erythrocyte

Sabyasachi Dasgupta [1], Thorsten Auth [1], Nir S. Gov [2,3], Timothy J. Satchwell [4], Eric Hanssen [5], Elizabeth S. Zuccala [6,7], David T. Riglar [6,7], Ashley M. Toye [4,8], Timo Betz [3], Jake Baum [6,7,9]* and Gerhard Gompper [1]*

DESCRIPTION OF ERYTHROCYTE-MEMBRANE WRAPPING

Towards calculating membrane-wrapping contributions to invasion we sought to identify core parameters associated with parasite entry. Four key parameters were selected, adhesive forces, bending energy, target membrane tension and a line tension (Fig. S1).

When the host membrane (Fig. S1*A*, orange) comes close to the parasite membrane (Fig. S1*A*, blue), adhesion molecules form an attractive interaction between the two membranes (illustrated with red springs). The adhesion strength, wH_0, which quantifies the adhesion energy, depends on the local concentration of the adhesion molecules. In experiments, close contact between the merozoite and the erythrocyte membrane is observed [1] that supports a close adhesive interaction between the two cells (Fig. S2*A*).

Bending a membrane also requires energy that is determined by two contributions; the bending modulus κ, which reflects the rigidity of the membrane against a forced bending, and the actual degree of bending which is quantified by the curvature C (Fig. S1*B*). The curvature C can be simply visualized by fitting a circle along the curve and then taking the inverse radius of this circle: $C = 1/R$. Hence, high curvature corresponds to a small circle radius, while a flat membrane has no curvature, which corresponds to an infinite radius. Since the membrane is a two-dimensional ($2D$) surface, curvature can exist at any point in the two directions. The relevant curvature is the mean curvature, $H = (1/R_x + 1/R_y)/2$.

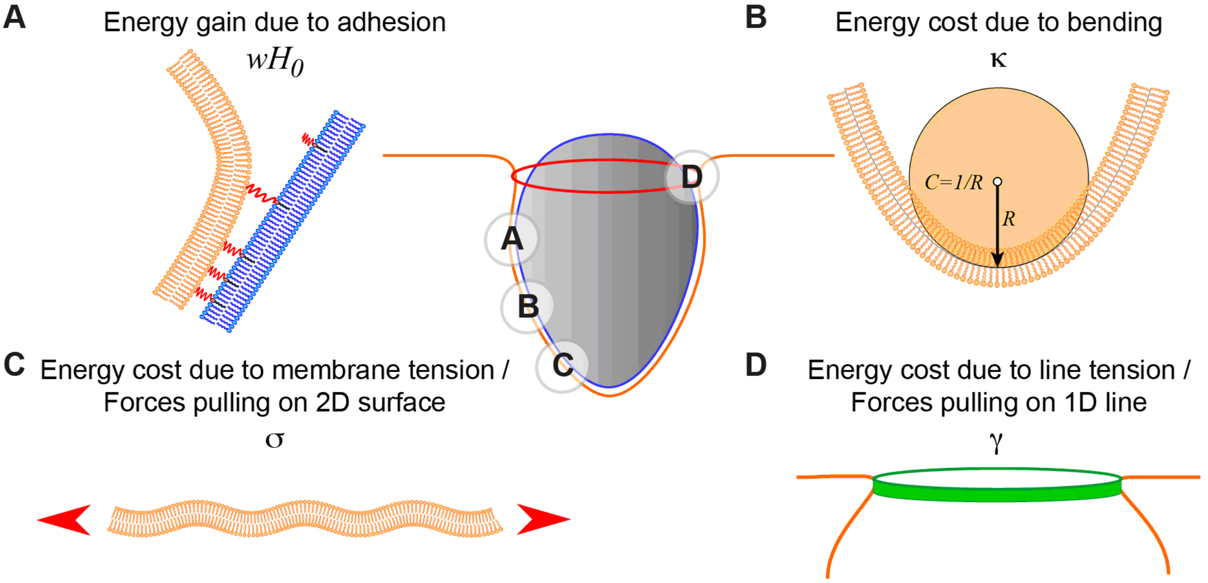

Figure S1. Schematic explanation of the energetic contributions used to model the role of the host membrane in merozoite invasion
The energetic contributions to membrane wrapping in our model are characterized by **A.** adhesion strength, **B.** bending rigidity**, C.** membrane tension, and **D.** line tension (see main text for details).

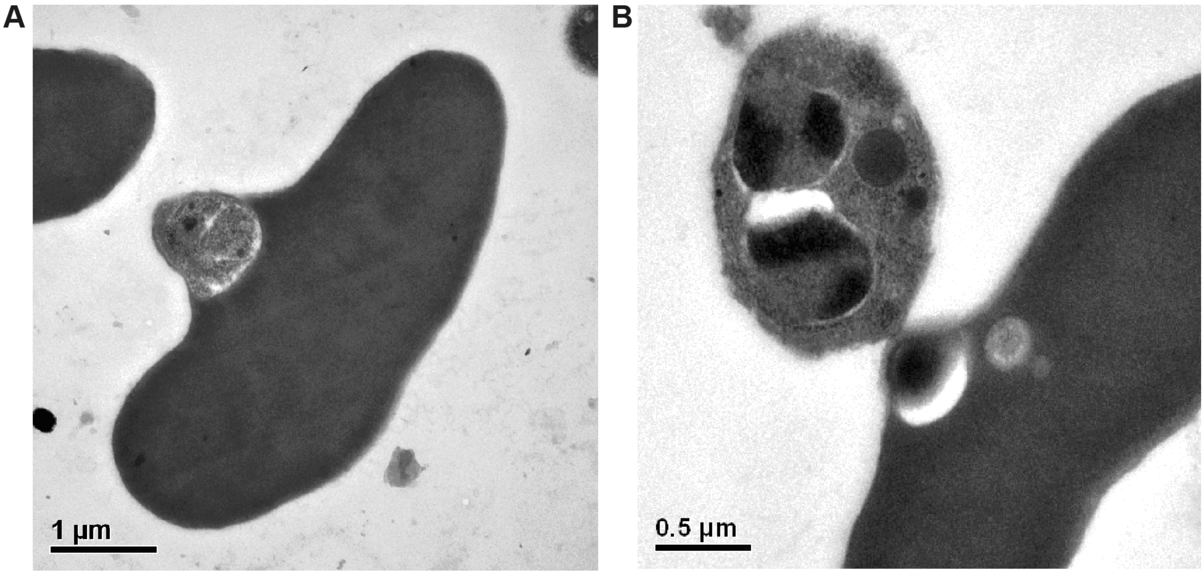

Figure S2. Electron micrographs of merozoites from *Plasmodium falciparum* in the process of invading an erythrocyte.
A. Demonstration of the close proximity between plasma membrane of the merozoite and the plasma membrane of the erythrocyte during invasion as revealed by high pressure freezing and freeze substitution (as compared with other approaches which can give rise to a substantial slack space between parasite and host cell in nascent parasitophorous vacuole). Imaging conditions as described in Ref. [1]. **B.** Release of membrane from apex of parasite during the invasion process. Here merozoite invasion is arrested with cytochalasin D (an inhibitor of invasion), which prevents completion of invasion but does not prevent release of membrane whorls from the rhoptry bulb. Imaging conditions as described in Ref. [1].

Two tension forces play key roles in the invasion process. Host membrane tension relates to an energetic cost that is related to a lateral pulling on the membrane (Fig. S1*C*). Typically, biological membranes are soft enough to fluctuate because of thermal movement, which is analogous to the Brownian diffusion of a small particle. This movement means that the apparent membrane area measured when looking on the membrane is smaller than the real area that includes the area “hidden” in the fluctuations. Physically, it means that when a pulling force is applied (red arrows), the apparent membrane area increases. The membrane tension can also be reduced by release of membrane from the apex of the parasite (Fig. S2*B*). The membrane tension is then defined by the force required to increase the membrane area. Hence high tension (represented by the term σ) means that high forces are required to increase the apparent area.

A final energetic contribution comes from the spring-like behaviour of the cytoskeleton along the furrow at the membrane inclusion, referred to here as a line tension (Fig. S1*D*). This spring-like behaviour can be modelled by an elastic rubber band that lies around the furrow, and hence wants to constrict the furrow. The spring stiffness of this rubber band is modelled by the line tension (represented by the term γ). Physically, in the first phase on invasion (PW I) the merozoite has to stretch this rubber band, which costs energy. However, once over the point of maximal diameter (PW II), the line tension actually helps pushing the merozoite inside the host cell.

CALCULATION OF THE MEROZOITE WRAPPING ENERGY

The key ingredients for calculating the energy for wrapping a merozoite are the shape of our archetypal merozoite and our model for the deformation energy defined by Eq. 2 of the main text. The egg shape of the archetypal merozoite is $(x^2+y^2+z^2)^2 = R_a x^3 + (R_a - R_b)x(y^2+z^2)$ with $R_a = 1\,\mu m$ and $R_b = 0.7\,\mu m$. For the calculation of the deformation energy, the shape is parametrized in spherical coordinates using the polar angle u and the azimuthal angle v,

$$x(u,v) = \left[\frac{(2R_a - R_b)\,sin\,(v)}{4} + \frac{R_b\,sin\,(2v)}{8}\right]\cos(u), \tag{1}$$

$$y(u,v) = \left[\frac{(2R_a - R_b)\,sin\,(v)}{4} + \frac{R_b\,sin\,(2v)}{8}\right]\sin(u),$$

$$z(u,v) = \left[\frac{2R_a + R_b\,cos\,(v)}{4}\right]\cos(v),$$

where $0 \le u < 2\pi$ and $0 \le v < \pi$. The distance from the symmetry axis as function of the azimuthal angle is

$$\rho(v) = \sqrt{x^2+y^2} = \left[\frac{(2R_a - R_b)\,sin\,(v)}{4} + \frac{R_b\,sin\,(2v)}{8}\right] \tag{2}$$

and the radial coordinate of the merozoite surface,

$$r(v) = \sqrt{x^2+y^2+z^2}$$
$$= \sqrt{\left[\frac{(2\,R_a - R_b)\,sin\,(v)}{4} + \frac{R_b\,sin\,(2v)}{8}\right]^2 + \left[\frac{2R_a + R_b\,cos\,(v)}{4}\right]^2} \tag{3}$$

In spherical coordinates, Eq. 2 in the main text becomes

$$\mathcal{E} = 2\pi\int_0^{v_{\mathrm{wrap}}} d\,v\,g_{\rho v}\,\rho(v)\,[2\kappa(H(v))^2 + \sigma - wH(v)] + 2\pi\rho(v)\gamma, \tag{4}$$

where $g_{\rho v} = \sqrt{(d\rho^2 + dv^2)} = (2R_a - R_b)\sin(v)/4 + R_b\sin(2v)/8$, and v_{wrap} determines the degree of wrapping. Note that in our model the membrane deformation energy is only calculated for the membrane that is attached to the merozoite. The integrals are evaluated numerically using the computer algebra program *Maple*.

Both the bending energy and the adhesion energy contributions in our model require the calculation of the mean curvature. The mean curvature can be expressed by the fundamental forms of the merozoite surface [2],

$$H = \frac{eG - 2fF - gE}{EG - F^2}, \tag{5}$$

where E, F, and G are the first fundamental forms and e, f, and g are the second fundamental forms.

DISCONTINUOUS PHASE TRANSITIONS WITH ENERGY BARRIERS

The phase boundary W_0 between the NW and the PW states in Fig. S3 and Fig. 4 in main text is the binding transition that we use to discuss reorientation based on the local adhesion strength and bending energy. With a small membrane and line tension, PW and even CW regions may extend to smaller adhesion strengths than those associated with the threshold adhesion strength for W_0. This might at first appear to be surprising, since for very shallow wrapping the value of the membrane tension would not be expected to alter the transition [3,4]. However, the nature of the W_0 transition and the W_1, W_2, and E transitions in the phase diagram are different: while the W_0 transition is a continuous transition, the transitions W_1, W_2 and E are associated with an energy barrier (E also indicates when the unwrapped and the completely-wrapped state have equal energy). These latter transitions correspond to a jump in the fraction of the merozoite surface that is in contact with the enveloping membrane as the transition is passed (Fig. 4). For adhesion strengths very close to those phase boundaries, transitions cannot occur spontaneously.

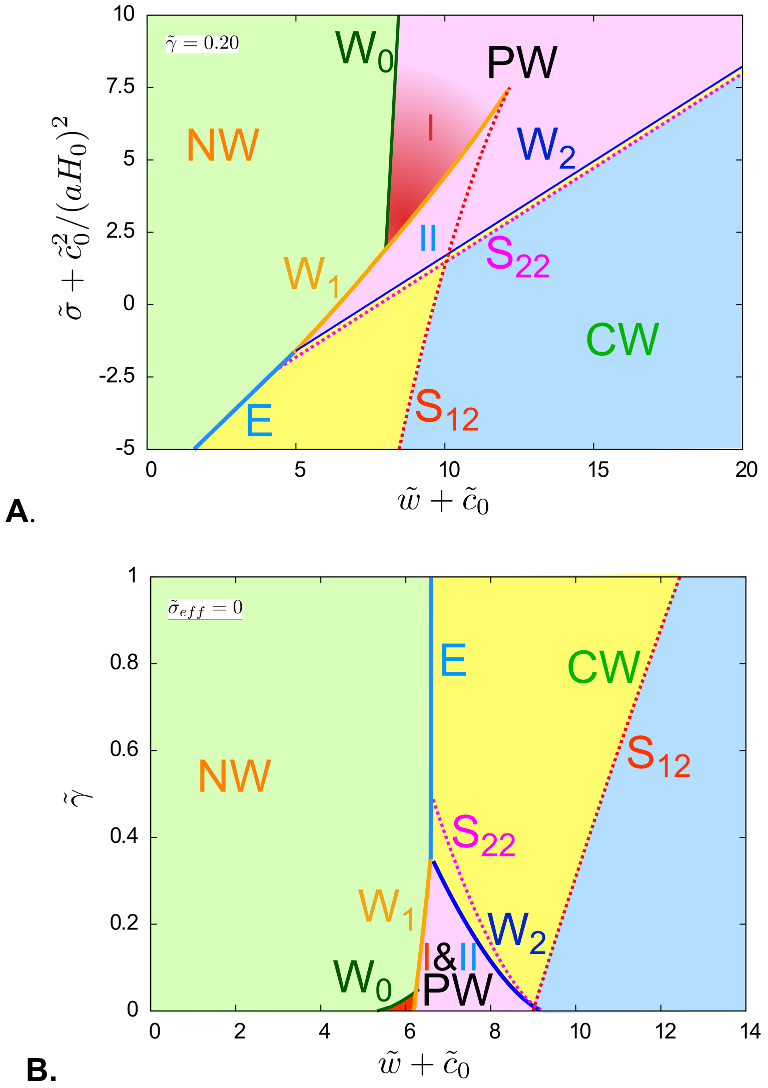

Figure S3. Phase diagrams for a tip-first oriented merozoite.
Phase diagrams Fig. 4 in the main text including the upper spinodals. S_{12} indicates the adhesion strengths at which the transition associated with the phase boundary W_1/E occurs spontaneously, while the spinodal S_{22} belongs to the phase boundary W_2. Spontaneous wrapping therefore occurs in the blue part of the CW region, while in the yellow part an energy barrier has to be overcome. **A.** Wrapping states for fixed reduced line tension $\tilde{\gamma} = 0.20$ and several values of adhesion strength and membrane tension. **B.** Wrapping states of the system of a tip-first oriented merozoite for vanishing effective membrane tension and several values of adhesion strength and line tension.

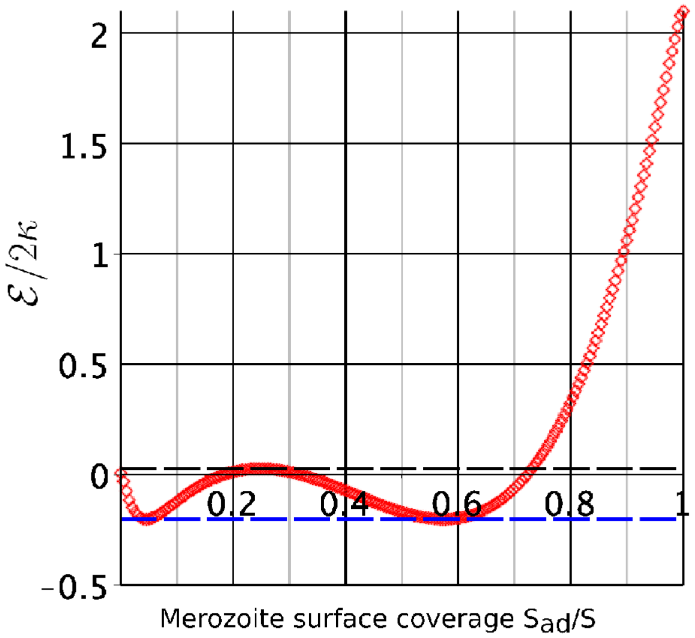

Figure S4. Energy profile for a state on the W1 phase boundary.
Energy profile at reduced effective adhesion strength $\tilde{\boldsymbol{w}}_{\boldsymbol{eff}} = \mathbf{10.066}$ and reduced membrane tension $\tilde{\boldsymbol{\sigma}} = \mathbf{4.5}$, see Figs. 4 and 5 in main text. The transition between the PW I state for $\boldsymbol{S_{ad}/S} = \mathbf{0.045}$ and the PW II state for $\boldsymbol{S_{ad}/S} = \mathbf{0.575}$ is associated with an energy barrier $\boldsymbol{\Delta\mathcal{E}/2\kappa} = \mathbf{0.228}$ whose maximum is found at $\boldsymbol{S_{ad}/S} \approx \mathbf{0.25}$.

For the phase boundaries W_1 and W_2, the spinodals S_{12} and S_{22} indicate the parameter regime where the transition occurs spontaneously and beyond that the energy barrier vanishes with increasing $\tilde{w} + \tilde{c}_0$. Only for values of adhesion strength that are larger than those of the spinodals does the energy barrier vanish and further wrapping occur spontaneously. In Fig. 5 in main text, we indicate the wrapping fractions of the stable states between which the discontinuous transitions occur; the tie lines mark the parameter region that corresponds to states on the energy barrier. In Fig. S4 we show the energy profile for a state on the discontinuous transition W1 when the system traverses from a low wrapping fraction (PW I) to a higher wrapping fraction (PW II). The profile shows explicitly the energy barrier that needs to be overcome for further wrapping at the adhesion strength $\tilde{w} = 10.066$ for reduced membrane tension $\tilde{\sigma} = 4.5$. The low wrapping fraction state is found at $S_{ad}/S = 0.045$ and the high wrapping fraction state at $S_{ad}/S = 0.575$. The maximum of the energy barrier with height $\Delta\mathcal{E}/2\kappa = 0.228$ is located at $S_{ad}/S \approx 0.25$. In order to jump from the PW I state to a PW II state, there is thus a need for an “activation energy” that facilitates invasion. The energy barrier for the W_2 transition that separates the partially-wrapped state with high wrapping fraction from the completely-wrapped state is typically much smaller than the energy barrier for the W_1 transition. Hence, the spinodal is found for adhesion strengths close to values where the W_2 transition occurs. Actomyosin motor activity may be a key determinant to overcome wrapping energy barriers and the barriers can be used to estimate the required motor activity. However, activity alone is not sufficient to study invasion. When the motor stops to act (e.g. once invasion is almost complete) the merozoite will only remain wrapped in cases where its final state lies within the CW region of the phase diagram (Fig. S3 and Fig. 4 in main text).

Supporting References

1. Hanssen E., Dekiwadia C., Riglar D. T., Rug M., Lemgruber L., Cowman A. F., Cyrklaff M., Kudryashev M., Frischknecht F., Baum J. and Ralph S. A. 2013. Electron tomography of Plasmodium falciparum merozoites reveals core cellular events that underpin erythrocyte invasion. *Cell Microbiol*. **15**: 1457-1472.
2. Elsa A., Salamon S. and Gray A. 2006. *Modern differential geometry of curves and surfaces with Mathematica*. CRC Press.
3. Lipowsky R. and Döbereiner H. G. 1998. Vesicles in contact with nanoparticles and colloids. *Europhysics Letters*. **43**: 219.
4. Farago O. and Pincus P. 2004. Statistical mechanics of bilayer membrane with a fixed projected area. *Journal of Chemical Physics*. **120**: 2934.
